# Mapping, characterisation, and analysis of initiatives with transformative capacity: A scoping review

**DOI:** 10.1007/s13280-025-02133-9

**Published:** 2025-02-14

**Authors:** Maíra Sardão, Pedro Gabriel Silva

**Affiliations:** Center for Transdisciplinary Studies for Development, CETRAD, University of Trás-os-Montes and Alto Douro, UTAD, Quinta de Prados, 5000-801 Vila Real, Portugal

**Keywords:** Intentional communities, Political ecology, Post-development, Resistance, Transformative initiatives, Transitions

## Abstract

The increasing complexity of socio-environmental vulnerabilities and inherent challenges have led scholars to study nonlinear systemic societal changes. Approaches to address these challenges range from adaptation to the predominant system, to the reconfiguration of the social and ecological life system. The study identifies the key characteristics, dynamics and contextual features of transformative initiatives and assesses their potential to challenge the dominant system. It also aims to review the studies of alternative non-capitalist experiences across a diverse geographic and cultural contexts. To achieve that purpose, a scoping review of empirical studies published since 2000 was conducted. The 88 cases analysed evidence practices rooted in cooperative and solidarity values. These practices include alternative approaches to production, consumption, and distribution, as well as the sharing of knowledge, experiences, and resources. The studies also point to the need to recreate and reorganise the commons, to change human and non-human interactions, and to achieve self-sufficiency.

**Supplementary Information:**

The online version contains supplementary material available at 10.1007/s13280-025-02133-9.

## Introduction

The scientific community has recently reached a consensus on the undeniable impact human activities have on climate systems, as reported by the IPCC (2021, 2022). According to the same source, those impacts have contributed to a rise in the number and frequency of extreme climate events and loss of biodiversity, imposing significant effects on land use. Furthermore, it evidences an unprecedented socio-ecological crisis marked by an unequal distribution of the impacts on human and non-human subjects (Acosta 2016; Scheidel et al. 2018; Kothari et al. 2019; Althor and Witt 2020).

Amid the swelling complexity of socio-environmental vulnerabilities, scholars have been steering an ample debate, looking into nonlinear systemic societal changes based on different methods and conceptual frameworks (Few et al. 2017; Sovacool and Hess 2017; Hölscher et al. 2018). Responses to the aforementioned vulnerabilities range from minor adaptations within the prevailing system to reconfigurations of social and ecological life. However, because incremental adaptations tend to reproduce the economic and political dynamics that prioritise capital accumulation and accept environmental degradation as a byproduct, they ultimately fail to prevent ongoing environmental damage (Peet et al. 2010; Few et al. 2017; Sovacool and Hess 2017; Benjaminsen and Svarstad 2018; Hölscher et al. 2018; Feola 2020; Felli 2021). This scoping review (ScR) focuses on the responses that seek to reconfigure social and ecological life. To do so, a review of empirical studies published in scientific databases was conducted (88 cases dating from 2000 onwards) aiming to identify initiatives and practices with transformative capacity. The ScR explored the practices, dynamics, and values (Few et al. 2017) adopted by agents in different territories while considering the structural contexts in which these initiatives dwell. The review converges with what Kothari et al. (2019) referred to as a *tapestry* of alternative (and) non-capitalist experiences (Burke and Shear 2014; Escobar 2015). The approach taken addresses key recommendations vented by Political Ecology authors such (i) synthesising a substantial body of research grounded in sustainability and environmental justice (Cavanagh and Benjaminsen 2017), (ii) establishing a structured relationship between concepts and real-world experiences (Robbins 2003) and (iii) crafting socio-environmental policies that, by embracing diverse political viewpoints, can include solutions other than exclusively technical ones (Castree 2002). Furthermore, this ScR feeds the transition’s debate by providing evidence of the factors that may influence changes, like those connected to the territory (Hansen and Coenen 2015).

For a more comprehensive grasp of the initiatives' potential to foster significant shifts in values and institutional or socio-economic structures (Escobar 2015), it is crucial to place them within the framework of the two primary positions that have been prominent in the debate: transition and transformation. Together, they offer various perspectives for explaining, analysing, and advocating social change. Despite their differences stemming from diverse origins and research groups, both approaches concentrate on changes occurring within complex adaptive systems (Feola 2015; Hölscher et al. 2018). Besides seeking comprehension of how “desirable radical and nonlinear societal change” (Hölscher et al. 2018, p. 2) takes place, both approaches also care to encourage that transformation.

The following section presents the specificities and convergences within the Societal Transformation framework (Feola 2015), followed by scholars' perspectives on transformative capacity and their definition of *alternative approaches* based on cultural and territorial aspects. Subsequently, after outlining the methodology, the findings are presented and discussed in the following two sections.

## Contextualising transformation and transition

In light of Feola's (2015) research on Societal Transformation, it has been observed that there is a lack of consensus regarding the conditions that promote transformational dynamics between the human and the environmental spheres. The author identified two main approaches covering, in an integrated manner, those dynamics: one, analytical-descriptive, and another, solution-oriented. The latter focuses on solving identified problems and prioritises applicable, transdisciplinary, and action-based methodologies. To this end, the social actors are seen as active participants crucial to achieving the necessary resolution, and the concepts can be broadened to accommodate diverse viewpoints. Conversely, due to its academic intent, the analytic-descriptive approach relies on precisely defined concepts to leverage researchers’ interpretations, comparisons, and debates. Within this framework, power relations and values are examined regarding their role in the process, thus facilitating an understanding of human-nature interaction (Feola 2015).

Drawing on the analytical-descriptive perspective outlined above, we can identify several factors that distinguish the concepts of transformation and transition. These factors include distinct epistemological underpinnings, diverse perspectives on how to define, interpret, and support societal changes, and even the etymological origins of the terms themselves (Hölscher et al. 2018; Schmid and Smith 2021).

In a comparative study of the concepts, Hölscher et al. (2018) identifies that transformation often revolves around a significant and widespread paradigm shift within society, with a particular emphasis on global environmental change. It involves fundamentally altering human and environmental interactions and yielding outcomes such as “safe and just operating spaces” (Hölscher et al. 2018, p. 2). The current research integrates perspectives centred on “alternative economies and political spaces” (Schmid and Smith 2021, p. 2), gathering studies on degrowth, post-capitalism, social and solidarity economy, transformative adaptation and other forms of transition discourses (TD)^1^ (Escobar 2015; Few et al. 2017; Kothari et al. 2019; Schmid and Smith 2021). As suggested by its etymology,—‘change in shape’—, analyses about transformation aim to identify the “emergent patterns of change” and their systemic outcomes (Hölscher et al. 2018, p. 2).

Conversely, as Hölscher et al. (2018, p. 1) claim, transition research looks at cultural, ecological, economic, institutional, or technological “change from one societal regime or dynamic equilibrium to another”. These transition research has been primarily applied to analyse *single systems* such as food, energy, mobility, and productive sectors, although it has also been used to study, in a comprehensive manner, the interconnections between such systems (Feola 2015; Loorbach et al. 2017; Hölscher et al. 2018; Köhler et al. 2019). Having sustainability transition (ST) as its prominent theoretical framework (Schmid and Smith 2021), transitional research has been focusing on explaining “‘how’ the nonlinear shift from one state to another is supported or hindered” (Hölscher et al. 2018, p. 2). The multidimensional and structural changes within ST render its analysis akin to a ‘puzzle’ (Geels 2011), allowing for the utilisation of various methods: Multi-level Perspective, Strategic Niche Management, Transition Management and Technological Innovation Systems (Schot and Geels 2008; Köhler et al. 2019; Schmid and Smith 2021). ST research shares a common concern, which varies in emphasis and analytical lens depending on the approach—Socio-technical, Socio-institutional, or Socio-ecological (Loorbach et al. 2017)—that is: “how can society support transitions to alternative social and economic systems, or embark on fundamentally different pathways to sustainability?” (Köhler et al. 2019, p. 22).

The core idea behind structural change, redesigning “modern society as a whole” (Feola 2015, p. 377), is present in the debates developed within both research strands. The concepts are not mutually exclusive despite their nuanced approaches and analytical tools for examining and supporting fundamental changes. Both recognise complex adaptive nonlinear systems as their object of study, and acknowledge uncertainties, heterogeneous values, and power struggles. Consequently, there is a shared recognition that the process entails ruptures and discontinuities (Feola 2015; Hölscher et al. 2018).

Societal Transformation acknowledges processes of adaptation and resilience. However, some authors consider adaptation a possible pathway towards transition, viewing it as a gradual adjustment process that may not necessarily trigger stress but rather leads to a co-evolution into a new regime (Hans de Haan and Rotmans 2011; Sovacool and Hess 2017). Conversely, others concentrate on the transformative capacity of adaptation, wherein the root causes of socio-ecological vulnerabilities are addressed (Few et al. 2017).

## Alternatives with transformative potentials

According to Kothari et al. (2019, p. xxxv), a societal shift can only be deemed ‘alternative’ if it encompasses at least “a potential for living change” without necessarily needing to be entirely radical. However, these alternative actions must challenge the capitalist logic rooted in wealth accumulation and economic growth. A variety of experiences, knowledge, or initiatives that aim for a profound and interconnected reconfiguration of human life and the biophysical environment, whether originating from the Global South or the Global North, can be identified. These endeavours can be assembled under the umbrella of TD, aiming for “fundamental change in values and novel socio-economic and institutions arrangements” (Escobar 2015, p. 453). However, drawing on anthropological evidence, Danowski and Viveiros de Castro suggest that such arrangements are not necessarily novel. These arrangements exist within some Amerindian ontologies, particularly evident in the inherently low material intensity ways of life practiced by certain indigenous populations (Danowski and Viveiros de Castro 2017), resonating with degrowth discourses.

In the Global North, TD tend to concentrate on degrowth and transition initiatives, while in the Global South they encompass alternatives to development or post-development proposals (Escobar 2015; Kothari et al. 2019; Garcia-Arias and Schöneberg 2021). A common demand articulated in these contexts is the recognition of biological and cultural diversity as well as of the interactions between humans, non-humans, and nature, thereby highlighting alternative socio-environmental arrangements (Leff 2001; Schlosberg 2007).

Faced with these multiple features, a reflexive discussion is needed. Firstly, transformative capacities should be examined and then a framework of analyses that considers the significance of territory within Societal Transformation debates should be elaborated.

### Reflecting on transformative initiatives

Descriptions and discussions of transformative initiatives can be found in academic literature across a broad spectrum of themes often intertwined with various conceptual approaches. Examples of contexts where the term ‘transformative initiatives’ is used include art (Rodriguez-Labajos 2022), technology (Beraldo and Milan 2019), future scenarios (Raudsepp-Hearne et al. 2020), knowledge (Roysen and Cruz 2020), rural-urban territories (Gorissen et al. 2018; Fèche et al. 2021), and energy systems (Atutxa et al. 2020). These studies draw upon, for instance, decolonial and feminist narratives (Rodriguez-Labajos 2022), sense of community (Unanue et al. 2020), grassroots innovation (Beraldo and Milan 2019; Roysen and Cruz 2020), and sustainability transition (Gorissen et al. 2018; Roysen and Cruz 2020).

From the perspective of these studies, it is feasible to piece together a comprehensive understanding of transformative initiatives as collective actions (Rodriguez-Labajos 2022; Atutxa et al. 2020) that engender transformative changes in social relations and society (Fèche et al. 2021) towards a “more sustainable, healthy and cooperative society” (Roysen and Cruz 2020, p. 978). These experiences must take into account biophysical limits and adopt a long-term perspective (Ross and Jones 2016).

The delineation of the concept of transformative initiative can also be discerned in its opposition to the prevailing hegemonic socio-economic model, as elucidated by the 85 essays about post-development analysed by Kothari et al. (2019). According to that study, such practices or worldviews prioritise the “*buen vivir* before material accumulation”, are based on cooperation rather than competition and “see work in pleasurable livelihoods, not ‘deadlihoods’ to escape from on weekends or ecotouristic vacations” (Kothari et al. 2019, p. xix).

Having identified the objectives and features proposed in the studies presented in this section, it is noticed that transformative initiatives aim for a radical shift that addresses the underlying causes of socio-environmental vulnerabilities, grounding solidarity values, relational logic, autonomy, and ecological consciousness (Dahle 2007; Kothari et al. 2019; Atutxa et al. 2020; Unanue et al. 2020). This approach may be summarised as: “to ground human activities in the rhythms and frames of nature, respecting the interconnected materiality of all that lives” (Kothari et al. 2019, p. xix).

To attain this objective, practices, experiences, and worldviews must feature certain transformative patterns or attributes. From a broader perspective, this capacity involves agency, interactions, and context. Specifically, different transformative capacities “refer to a multifaceted and emergent property of complex human-environment systems that relies on interdependent characteristics of agency and structure” (Wolfram 2016, p. 125). While focused on urban scenarios, but applicable more broadly, the author and fellow colleagues underscore the necessity for a simultaneous process of disrupting the prevailing system and constructing alternatives to it (Wolfram et al. 2019). Despite the challenges it presents, the current emphasis is on delineating the features, modes of action and practices with transformative potential.

### Adopting an analyses framework for transformative variables

Hence, how can one discern whether a particular practice, initiative, or experience has transformative potential? Drawing on the framework proposed by Few et al. (2017), one can elucidate the nature and intensity of the transformation capacity. The authors present a framework that pertains to significant alterations in response to climate change adaptation. According to their concept of “mechanisms of change”, initiatives can be classified in four distinct categories: Innovation, Expansion, Reorganisation, and Reorientation. While the former two are associated “with technical or behavioural adaptation actions”, the latter pair focuses on fostering changes in social structures. The divergence lies in their primary focus: the Reorganisation category is centred on governance structures, whereas the Reorientation category emphasises the transformation of “social values and social relations” (Few et al. 2017, p. 4). As outlined by the authors (2017, p. 5), the shift of dominant systems or structures can “be either abrupt and overt, or quite gradual and subtle in operation through a process of empowerment and negotiation”.

The aforementioned variable will be adapted to the context of alternative non-capitalist experiences, in order to identify the initiatives and practices with transformative capacity documented in the academic literature. Part of the challenge is to examine how territory influences these transition processes (Hansen and Coenen 2015).

### Locating the space and place within the societal transformation

Understanding geographical space is crucial for contextualising the discussion about a radical societal shift towards sustainability. Harvey (as cited in Miller 1998) argues that our conception of space should be fluid, including the map location (i.e. “situatedness”), the interplay of material and symbolic practices within that space, the power relations and our broader understanding of spatiality itself. Moreover, it is in place as a dynamic space, with its unique characteristics and global connections, that the forces of globalisation and fragmentation intersect, creating new possibilities and constructing futures (Santos 1996).

Drawing on the work published a decade before by Nicholls and Beaumont, Nicolosi and Feola (2016) stated that there are two main perspectives for understanding geographic space. One perspective views space with distinct, clear-cut boundaries, while the other emphasises a relational approach, critiquing oversimplified dichotomies like *global vs. local*. By finding a middle ground between these perspectives, two key aspects can be seen: first, a purely relational focus can neglect the unique qualities of specific places; second, some relationships and dynamics transcend local contexts and specificities. As Hansen and Coenen (2015) suggest, it is important to understand how these seemingly opposing views can coexist, acknowledging their simultaneous influence and how they adapt across different scales and spaces.

Understanding the dynamics between agents, the interplay of formal and informal localised institutions, and the conditions and interactions that enable or hinder potential change are essential for laying the groundwork for a geography of transitions (Hansen and Coenen 2015; Köhler et al. 2019). This specific agenda of sustainability transition (ST) focuses on place-based features to explore and comprehend nonlinear systemic societal changes. It also reveals the spatial variations of “different transition processes” (Hansen and Coenen 2015, p. 4). One key element of this analysis is geographical proximity. It allows observing the formation of networks, alliances or conflicts, as well as access to resources and the dynamics of trust (Hansen and Coenen 2015; Feola and Jaworska 2018). The interactions between actors themselves, and between them and the elements of the territory (political-institutional or environmental) can be both extensive and intensive. This can lead to varying levels of trust intensity and different types of resources obtained. While intensive localised interaction fosters greater engagement and knowledge exchange between the groups involved, extensive relations tend to facilitate access to resources that are less dependent on geographic or social proximity, e.g. financial resources and political support (Nicolosi and Feola 2016).

The spatial perspective can further reveal the dynamics of subalternity within and across territories and their populations (Alimonda 2011). It serves as a key tool for recognising how space posits itself in the global political-economic scenario and for identifying their “experiences of marginalisation” (Galviz et al. 2022, p. 10). These experiences can be expressed through acts of resistance or inspiration (Radcliffe 2015) for broader societal change. These spaces can be seen as ‘alternative’ or ‘marginal’, when one observes their “diversity of alterity” (Longhurst 2013, p. 2100). Embodying a plurality of experiences, perspectives and perceptions, these spaces and the people living in it hold an ambivalent relationship with the centre: marginal or excluded from the dominant system, yet able to criticise it and influence societal transformations (Shields 1991).

In this regard, it is essential observe if the space provides a safe environment to nurture transformative initiatives, projects, or practices. From a broader perspective, these secure-spaces encompass five key characteristics: 1. recognition of an unsustainable and in need of change “dominant system and status quo”; 2. presence of actors empowered to drive “sustainability and social justice”; 3. ability to connect and integrate diverse actors, insights, and practices for transformation; 4. readiness to seize emerging or existing opportunities; 5. embracing the “creative tension” between diverse points of view (within the space) which may foster transformative change (Pereira et al. 2018, p. 2).

## Methods

This article was designed to map studies that explore knowledge, initiatives, or experiences with transformative capacity towards more sustainable ways of life grounded in socio-ecological approaches. The decision to conduct a scoping review was made because it provides researchers with a method to effectively manage a broad range of data, encompassing various natures, contexts and concepts (Arksey and O’Malley 2005; Tricco et al. 2018). The data were summarised and reported according to the Preferred Reporting Items for Systematic Reviews and Meta-Analyses extension for Scoping Reviews (PRISMA-ScR) statement (Tricco et al. 2018).

To address the research’s central question “What types of initiatives with transformative capacity, focused on a socio-ecological approach, can be identified and analysed in scientific indexed databases?”, a search string was developed to cover the terminology akin to ‘transition discourses’ (TD). The key terms and concepts to post-development and TD, as outlined by Kothari et al. (2019) and Escobar (2015), respectively, were initially tested independently in the Scopus and SciELO databases. The purpose was to ascertain which terms or concepts might be eligible for inclusion in the ScR. At this stage, the primary eligibility criteria was a focus on political ecology, environmental justice and decoloniality. The resulting search string was used on Scopus, Web of Science and SciELO databases, considering publications from the year 2000 onwards (Table 1). In terms of relevance and quality, the majority of the articles were published in first and second quartile journals, accounting for 68% and 11%, respectively. The remaining articles, although published in third and fourth quartile journals, were still included due to their alignment with the research scope. Additionally, the selection of articles and the variety of databases enabled the identification of empirical studies from diverse geographic contexts, ensuring significant representativeness. The initial search yielded 8.545 documents which were then reduced to 5.682 entries after eliminating duplicates using Mendeley software and the Rayyan web-based platform.

**Table 1:** Search keywords and data delimitation

String 1	(( “buen vivir” OR “liv* well” OR “good liv*” OR “good life” OR “suma* kawsay” OR “kume mongen” OR “suma qamana” OR “bio-civili*ation” OR “bio civili*ation” OR zapatis* OR ubuntu OR ukama OR postdevelopment OR “post-desarrollo” OR “post-development” OR “post-capitalis*” OR postcapitalis* OR “non-capitalis*” OR noncapitalis* OR *biocentr* OR “post-extractivis*” OR postextractivis* OR degrowth OR “post-growth” OR postgrowth OR “post-materialis*” OR postmaterialis* OR ecosocialism OR “eco-socialism” OR ecofeminism* OR “eco-feminism*” OR permaculture OR “transition initiative*” OR pluriverse* OR “transformative initiative*” OR “transition* discourse*” OR “alternative ways of living” OR “solidarity econom*” OR “mino bimaadiziwin” OR convivial* OR “eco-knowledge” OR ecovillage* OR neohippie* OR “neo-hippie*” OR “modern-hippie*” OR “modern hippie*” OR “neo-rural*” OR neorural* OR neorrural* OR counterurbani*ation OR deurbani*ation OR “desirable future*” OR bioregionalism* OR “bio-regionalism*” ) AND ( “environmental *justice” OR “political ecology” OR alternative* OR transformat* OR decoloni* OR postcolonial* OR “post- colonial*” ) )
String 2	((agroecolog* OR regenerat* OR “post-human*” OR posthuman* OR ecosocial OR “eco-social” OR “non-tradition* community” OR “re-locali*ation” OR relocali*ation) AND (“environmental *justice” OR “political ecology” OR decoloni* OR postcolonial* OR “post-colonial*”))
Databases	Scopus, Web of Science and SciElo
Limits	2000–2023 [last search done on March 27th]

**Fig. 1:**
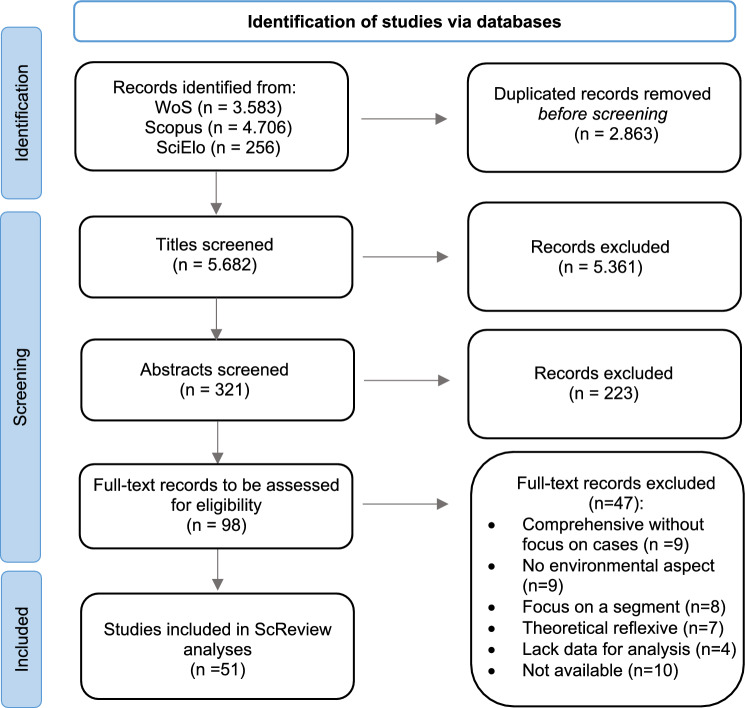
Scoping review flow diagram adapted from (Tricco et al. 2018; Page et al. 2021)

The screening process occurred in three different stages: title analyses (*n* = 5.682) followed by the abstract (*n* = 321) and full-text (*n* = 98) reading (Fig. 1).

In the two initial stages, the Rayyan platform was used. This web-based platform employs software to support screening and provides a collaborative work environment that enables independent blind revisions. The review was organised according to the criteria developed by Feola (2015) to address the studies on Societal Transformation. The documents were analysed by two reviewers following inclusion and exclusion criteria adapted to this study, discarding contents that (i) focus on the biophysical systems or infrastructure transformations (e.g. change on the ecosystem, landscape, or transport modes); (ii) address transformation in organisational environments (e.g. corporate change or actions in the context of education or health); (iii) restrict transformation to adaptation, rather than considering ruptures or structural changes (e.g. studies on instruments for measuring future scenarios or macroeconomic instruments focused on economic growth or changes in consumption patterns). The different perspectives resulting from the selection process were integrated by the two reviewers considering the above-mentioned criteria. Ultimately, the selection was narrowed down to 321 documents, including articles, books, chapters, and conference papers. The resulting materials laid the foundation for the next two screening stages: abstract reading followed by full-text analysis^2^.

### Data charting and summarising

To capture a broad range of documented experiences and initiatives, data from the full-text reading stage were summarised according to pre-defined variables. These variables, identified during the screening process were aligned with terms discussed in “Alternatives with transformative potentials” section. Using an Excel spreadsheet, the data were organised by author, year of publication, methodology and empirical study details. This included indexing information on location, project start year, main practices, actors involved, networks, and outcomes. The first researcher charted and summarised the data, which was then discussed with the second researcher for accuracy (Arksey and O’Malley 2005; Tricco et al. 2018).

### Synthesis of results

The information collected from the studies selected for the ScR is presented in a descriptive-narrative format in the following section. Key findings are also summarised visually using Tableau software and Excel (Tricco et al. 2018). Following a qualitative approach, the findings are segmented into three categories: (i) “Mechanisms of change” (Few et al. 2017), (ii) “Territory relation” (Feola and Jaworska 2018), and (iii) “Transformative space” (Pereira et al. 2018) (further details are presented in the previous section). The first category composes the variables adopted to identify the transformative potential of initiatives. The last two categories help us understand how the territory shapes the change process.

### Study limitations

This synthesis aims to provide a comprehensive overview of the characteristics and scope of the transformative initiatives across various territories. However, the breadth of the reviewed experiences presents two potential limitations. First, because priority was given to gathering as much studies as possible from different geographic contexts, not all studies were published in top-rated journals, despite consulting only indexed database. Second, even a comprehensive review of empirical studies cannot capture the full spectrum of existing experiences and practices. This limitation is particularly evident in the lack of literature on initiatives in Africa, Asia, and Oceania. To address this gap, further studies could broaden their scope by exploring other databases and incorporating other keywords in the search string, including languages beyond English, Spanish and Portuguese.

## Findings

This section presents the results in a descriptive-narrative format, structured in three subsections: (i) an overview of the study’s object, including the type of initiative, its geographical location and starting year; (ii) an identification and grouping of the initiatives according to five types of changes they were able to instil; and (iii) a classification and grouping of the interactions between agents within their territories taking into account existing networks and spatial conditions for transformation.

### Scope of transformative initiatives: type, location, and lifetime

**Fig. 2:**
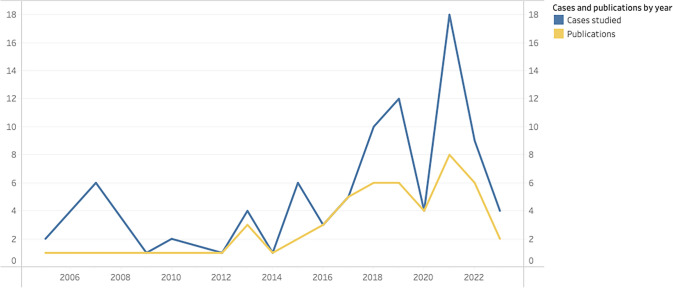
Case studies and publications by year

The selected 51 documents—including 16 articles and 3 books that used comparative or multiple study designs—provided 88 empirical cases for analysis (Fig. 2).

Regarding the subject of analysis, intentional communities (*n* = 49) were the focus of interest of the empirical studies included in the present ScR research. These communities, formed by people who share a common vision of life and pursue collectively, were categorised into ecovillages or villages (*n* = 36) and urban collective settlements (*n* = 13)—such as squatting and co-housing initiatives. The second category of initiatives considered the experiences of indigenous’ and peasants’ communities (*n* = 20), encompassing movements, settlements, and various forms of communal living. The remaining studies examined initiatives such as (i) cooperatives and associations (*n* = 8), e.g. Centre for Sustainable Agriculture (CSA); (ii) diverse projects (*n* = 7) focusing on food systems, energy solutions, and educational projects not restricted to single sector or organisational environments; and (iii) alternative currency networks (*n* = 4).

**Fig. 3:**
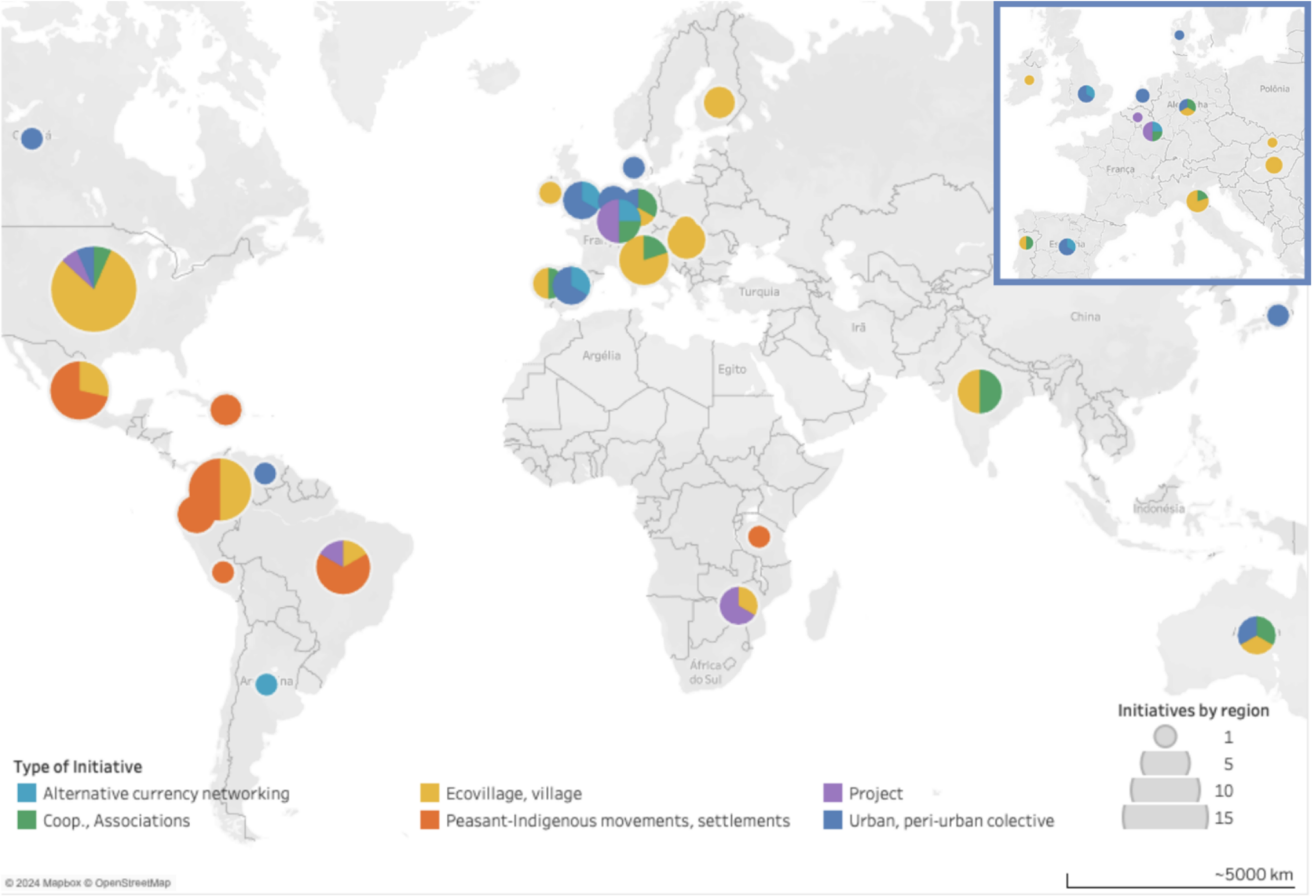
Country-level distribution of case studies by initiative type

Most of the cases analysed were in the USA, followed by three Latin American countries—Colombia (*n* = 8), Mexico (*n* = 7) and Brazil (*n* = 6)—and one Mediterranean country—Italy (*n* = 5) (Fig. 3). All five continents were included in the analysis (Table 2). The American continent was the primary site of the experiences studied, 45 in total (in North America, the USA alone accounted for 15 cases, while in Latin America had 29 cases spread across 8 countries). Europe followed with 31 cases from 13 countries. Oceania was represented by only one country (Australia, *n* = 3), the African and Asian continents were represented by two countries each: Zimbabwe (*n* = 3) and Tanzania (*n* = 1), and India (*n* = 4) and Japan (*n* = 1).

**Table 2:**
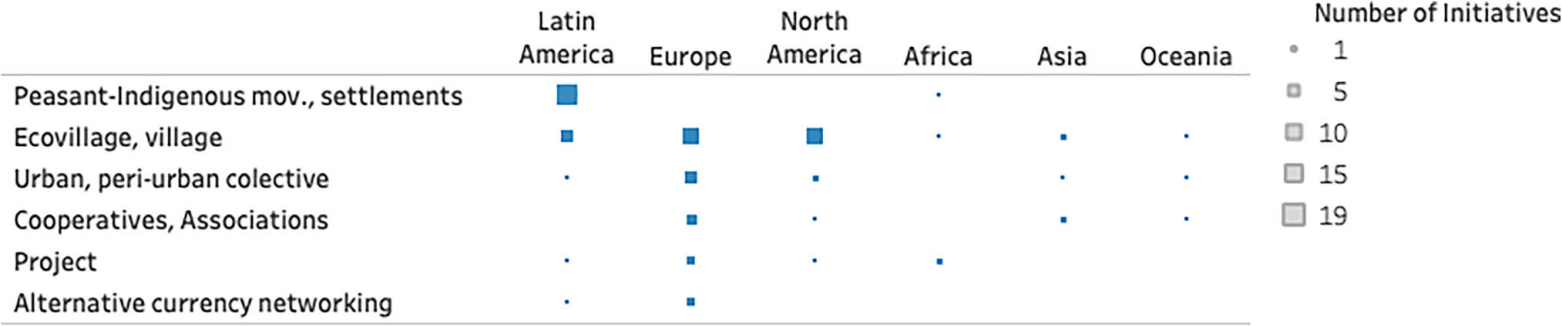
Geographic variation in initiatives types across continents

**Fig. 4:**
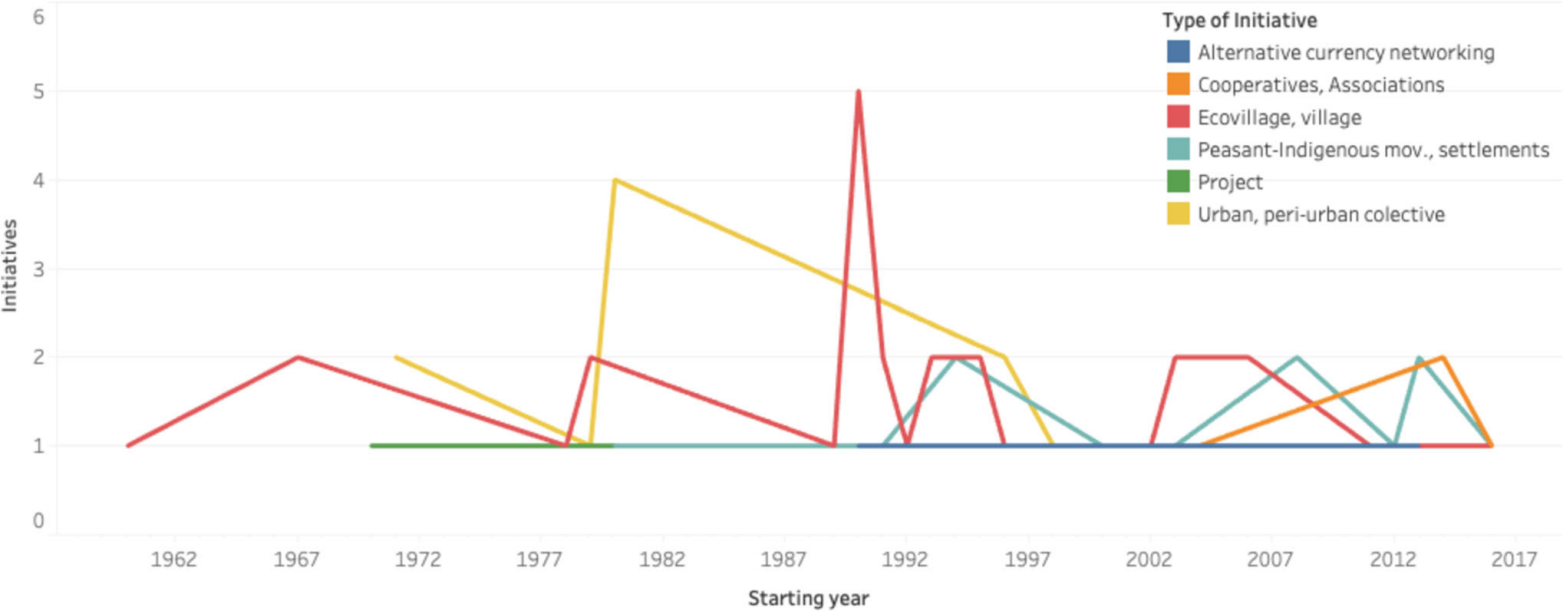
Temporal distribution of initiative start years by type (*n* = 75). Initiatives with unspecified star year and the 1895 initiative were excluded for visualisation purposes

Among the 76 experiences which reported their starting year, 35,5% saw the light of the day in the 1990s while 39,5% began after 2000. The remaining experiences started primarily in the 1970s (*n* = 7) and 1980s (*n* = 8), with a few additional ones emerging in 1960s (*n* = 3) (Fig. 4). The analysis also includes some of the longest-standing experiences such as the Norco Dairy cooperative in Australia (established in 1895), the Auroville in India (1960s) and the Twin Oaks intentional community in the USA (1967). The most recent initiatives studied began in 2016: the Territorio Campesino Agroalimentario, in Colombia, and the alternative food network SoLaWa, in Luxembourg.

### Mechanisms of change across different types of transformative initiatives

**Fig. 5:**
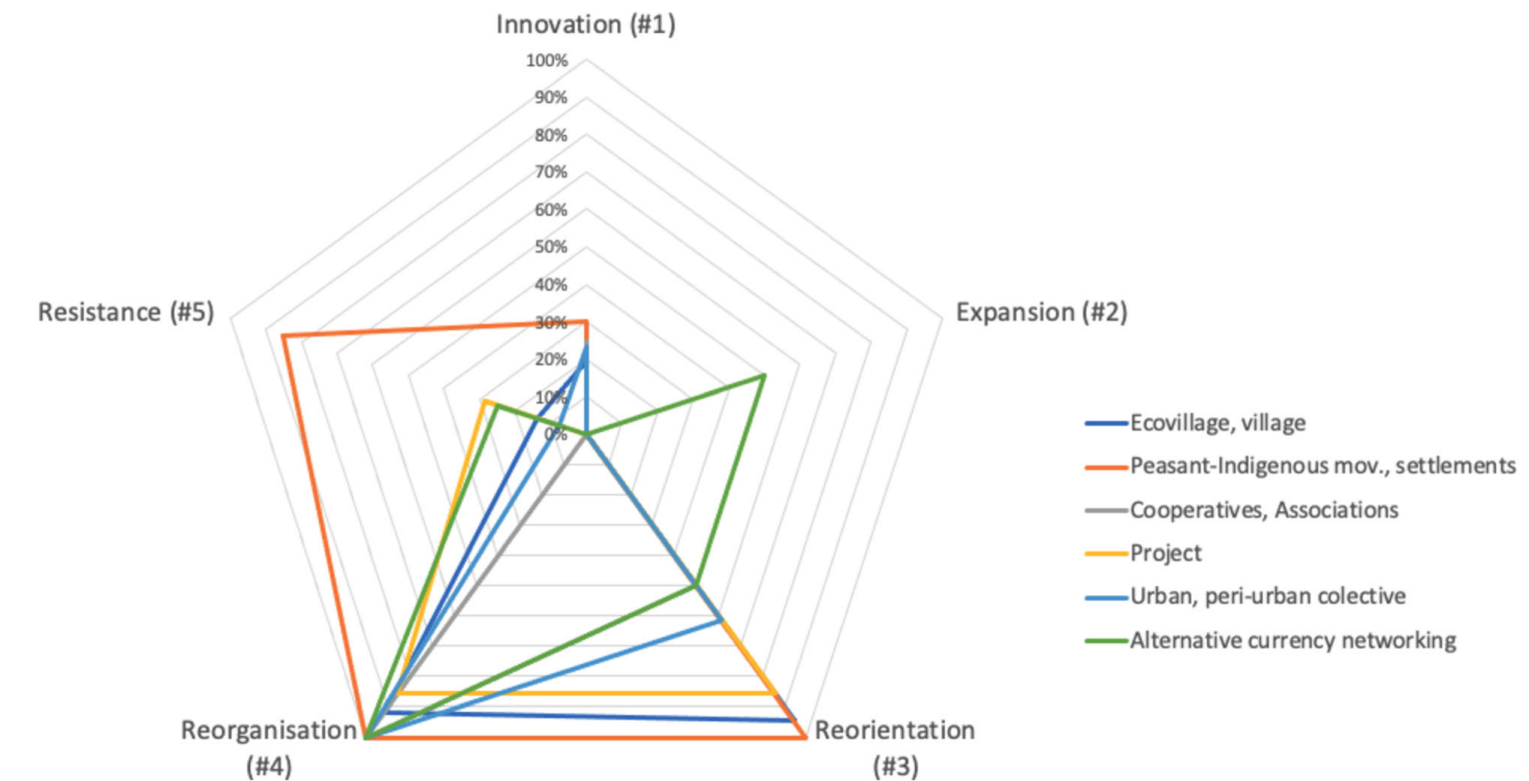
Mechanisms of change observed in studies (*n* = 88): Adaptation and Transformation. The first two mechanisms (1. Innovation; 2. Expansion) involve adaptation through techniques or behaviours. The remaining three (3. Reorientation, 4. Reorganisation, 5. Resistance) focus on transformative changes (adapted from Few et al. 2017)

A crucial aspect of the present ScR study involves identifying the transformative potential of practices, initiatives, and experiences. To grasp it, the framework proposed by Few et al. (2017) was adapted to weigh the initiatives’ capacity to promote social structural changes. This involved examining the mechanisms employed by the experiences. From a broad perspective, 72,2% of ecovillages have demonstrated ability to Reorganise their governance structures and Reorient individual and collective values—referred here as “2 Re” (Table 3 and Fig. 5). Regarding the peasant and indigenous movements, 15% of the experiences studied were also understood as 2 Re; however, in addition to the variables of Reorganisation and Reorientation, the strategies present in 60% of the other cases evidence self-determination, alterity, and defiance approach. Therefore, a Resistance variable was added to Few et al.’s framework. The remaining types of initiatives (36.4%) demonstrated a more diversified interplay of mechanisms of change. These included patterns of adjustment or adaptations driven by innovation and expansion processes that could be either connected to or independent of more fundamental structural changes.

**Table 3:** Type of initiative categorised by the sum of each variable related to the mechanism of change (*n* = 88) (adapted from Few et al. 2017). Most initiatives exhibit multiple change mechanisms. See the Supplement for a detailed breakdown of the variables associated with each one

Type of initiative	Total	Innovation (#1)	Expansion (#2)	Reorientation (#3)	Reorganisation (#4)	Resistance (#5)
Ecovillage, village	36	7	0	34	33	5
Peasant-Indigenous movements, settlements	20	6	0	20	20	17
Urban, peri-urban collective	13	3	0	8	13	1
Cooperatives, Associations	8	2	0	4	8	0
Project	7	0	0	6	6	2
Alternative currency networking	4	0	2	2	4	1
Total	88	18	2	74	84	26

### Characterising the influence of the territory on societal transformation

**Fig. 6:**
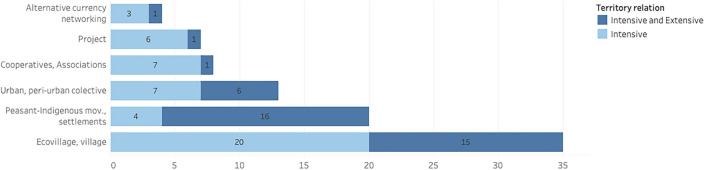
The interactions of the actors among themselves and between them and the elements of the territory were classified as intensive or intensive and extensive, based on the analysis of the selected empirical studies (*n* = 87) (Nicolosi and Feola 2016)

When observing the interactions among the agents in their territories, peasant-indigenous experiences tend to exhibit a prevalence of intensive and extensive networks simultaneously (80%), while exclusively intensive connections stand for 20% of the cases (Fig. 6). In contrast, ecovillages primarily rely on intensive relations (57%) but also establish broader geographical interactions (43%). Urban collective experiences show a slightly higher occurrence of intensive relationships compared to the combined approach. In turn, cooperatives and projects tend to function primarily based on local proximity-based connections.

**Fig. 7:**
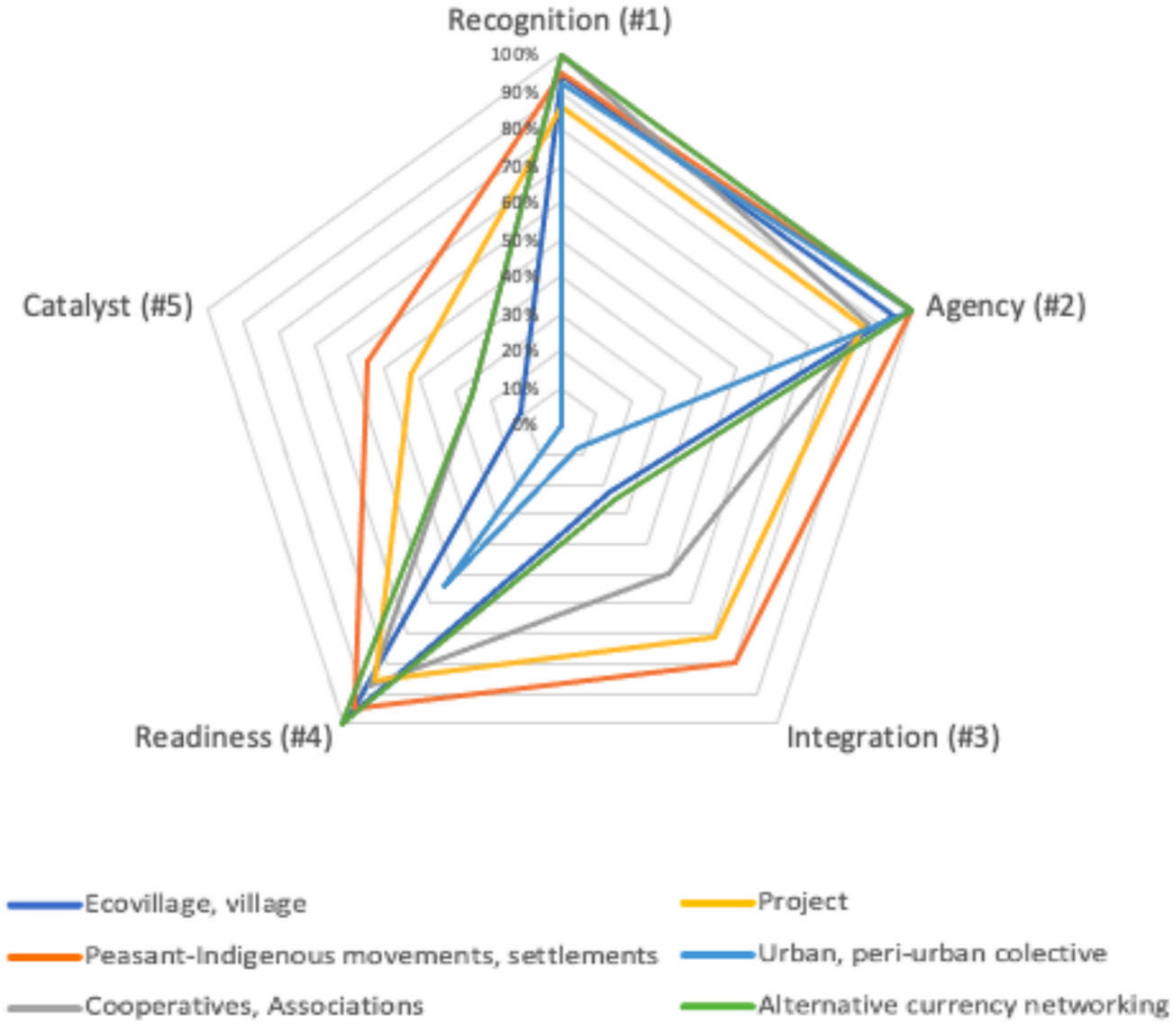
Territorial-based characteristics with a potential to foster transformation observed on the studies (*n* = 88): 1. recognition of need to change the dominant system; 2. presence of mobilizing agents; 3. ability to integrate diverse actors; 4. readiness to seize opportunities; 5. Fuzing diverse worldviews as catalyst of transformative changes (based on Pereira et al. 2018)

In terms of space characteristics, considering the conditions to let transformations foster (Pereira et al. 2018), the analysis identified two main scenarios represented by the groupings 1.2.4 (*n* = 36) and 1.2.3.4.5 (*n* = 18) (see Supplement for details). Take ecovillages, for example, which fall into the first grouping (1.2.4): their members often identify unsustainable patterns and the need to provide a safe space for challenging the *status quo* (characteristic 1) involving people with agency (characteristic 2) and with conditions to prepare themselves to deal with future or current opportunities for change (characteristic 4) (*n* = 23 cases). However, the capacity to involve heterogeneous agents (characteristic 3) and to create innovative solutions from emerging tensions (characteristic 5) were not observed in these types of initiatives (with the exception of three cases). In contrast, all five characteristics were identified in half of the peasant-indigenous experiences, suggesting that they might functions as “transformative space”. Conversely, 54% of the cases classified as urban collective have evidenced a space with fewer features to stimulate change. Looking at the big picture (as shown in Table 4), different types of initiatives tend to exhibit all five characteristics, but in varying proportions that either enable or hinder transformation within their territories (as illustrated in Fig. 7).

**Table 4:** Type of initiative by the sum of each variable related to the territorial-based characteristics with a potential to foster transformation observed on the studies (*n* = 88) (based on Pereira et al. 2018)

Type of initiative	Total	Recognition (#1)	Agency (#2)	Integration (#3)	Readiness (#4)	Catalyst (#5)
Ecovillage, village	36	34	34	8	35	4
Peasant-Ind. mov., settlements	20	19	20	16	19	11
Urban, collective	13	12	13	1	7	0
Cooperatives, associations	8	8	7	4	7	2
Project	7	6	6	5	6	3
Alternative currency networking	4	4	4	1	4	1
Total	88	83	84	35	78	21

## Discussion: plural perspectives and alternatives

This scoping review aimed to identify the types of initiatives documented in scientific databases with potential to challenge the dominant system. The main findings of the ScR are discussed in this section based on a threefold perspective. First, drawing on the framework of Few et al. (2017), the analysis emphasises on “emergent patterns” within the initiatives that can promote changes (Hölscher et al. 2018, p. 2). Second, an observation of the documented practices that indicate a shift in the values, governance, or intentions of the agents allows exploring the types of relations (intensive/extensive) existing within the territory (Nicolosi and Feola 2016), and the place-based elements that can foster transformative processes (Pereira et al. 2018). Third, the cultural nuances of the initiatives, complemented by the limitations and impacts of their practices, highlight strategies that can be translated into policy recommendations.

### The 3Rs of transformation: reorientation, reorganisation and resistance

Aligning the analysis of the cases with the Reorientation variable (Few et al. 2017) reveals a set of key practices related to the change of social values and relations, as condensed on Table 5 (see Supplement for details about each code). The analysis of the agents' values, behaviours, and priorities suggests a reorientation of social relations. This process represents a shift in subjectivity. The agents, mostly members of intentional communities and peasant or indigenous movements, develop subjectivity based on collective responsibility, reciprocity, and mutual aid. This is evidenced by their labour practices, daily routines and even by their communal political approach and subjectivity. Changes in community integration arise, as they move away from human-centred perspective and challenge the dominant capitalist patterns of ecological and social integration. In other words, the non-human perspective is also integrated in their worldview considering its flows and rhythms.

As shown by Few et al. (2017, p.4), the change in values and social relations can be understood as a “social learning” process along the journey towards transformation. Educational activities and knowledge sharing contribute to strengthen this journey. Despite the heterogeneity of experiences and initiatives, a few recurrent topics emerge, such as agroecology, lifestyle of intentional communities, conflict resolution skills, and local or ancestral knowledge, among others (Table 5).

**Table 5:** Reorientation in transformative initiatives. The agents’ changes in social values and behaviours are expressed through shifts in subjectivity and knowledge-sharing practices (see Supplement for details about each code)

Main practice	Specific practice	Codes
Shift in subjectivity	Adopting cooperative and solidarity-based values and behaviours in daily life and labour relations	2, 4, 5, 8, 12, 13, 14, 15, 20, 21, 23, 25, 29, 30, 31, 32, 34, 37a.b.d, 39, 51
	Incorporating collective political approach and subjectivity	14, 21, 27, 30, 35, 37b.c., 39
	Changing human/non-human interactions by incorporating the non-human perspective and the nature rhythms into their worldviews	3, 14, 18, 20, 23, 29, 30, 33, 34, 35, 37b.c.d, 38, 40
	Pursuing self-sufficiency and autonomy through alternative lifestyles and creative designs	1, 10, 18, 19, 22, 23, 24, 25, 29, 34a, 41, 50, 51
	Embracing a spatial-cultural-spiritual reconnection	1, 16, 18
“Social learning” in the context of educational activities and knowledge sharing	Engaging in agroecological knowledge and practices	5, 13, 15, 18, 19, 20, 23, 27, 30, 33, 35, 39, 48, 51
	Learning about and promoting intentional community lifestyles and self-sufficiency	1, 10, 11, 19, 22, 23, 24, 25, 28, 29, 41, 50, 51
	Developing new skills to deal with affection and emotional regulation and address internal conflicts	1, 11, 12, 20, 22
	Engaging in conflict resolution practices within the community and beyond	20, 21, 51
	Promoting equality and addressing gender relations	1, 11, 12, 14, 21, 23a.b, 30, 35, 37d, 39, 51
	Sharing and preserving local or ancestral knowledge	3, 16, 23, 29, 35, 37b.c.d, 39, 40, 48
	Utilising storytelling and music for knowledge transmission and cultural expression	5, 35, 37a, 39, 48
	Encouraging sociocultural and political debates	39, 47, 51

In three quarters of the analysed cases (*n* = 70), the reorientation and reconfiguration of values and behaviours are associated with changes in governance. It means that the initiatives also represent a Reorganisation in terms of practices, processes, mechanisms, arrangements, or structures, whether formal or informal, that are able to limit or reinforce certain understandings and choices regarding changes (Few et al. 2017). In general, studies emphasising this variable highlight efforts to (i) implement alternative production, consumption, and distribution systems, (ii) reclaim and reorganise the commons, (iii) collectivise deliberative processes, and (iv) engage in political acts and advocacy (Table 6).

**Table 6:** Reorganisation in transformative initiatives. The agents’ changes in governance are expressed through their practices related to economic systems, the commons, deliberative processes, and advocacy (see Supplement for details about each code)

Main topic	Specific practice	Codes
Alternative systems	Implementing alternative production systems (agroecology, permaculture, agroforestry, etc.)	2, 3, 5, 6, 8, 9, 11, 12, 13, 14, 16, 17, 18, 19, 20, 23, 24, 27, 29, 30, 33, 34a, 35, 37b, 39, 40, 43, 44, 45, 47, 48, 49, 50, 51
	Developing alternative consumption and distribution systems (local markets, producer-consumer relationships)	5, 6, 14, 16, 24, 26, 30, 33, 43, 45, 47, 51
	Adopting cooperative-based and hybrid economic practices (mixing market, solidarity economy, community currency, collective redistribution of earnings, etc.)	3, 4, 5, 8, 13, 14, 18, 21, 24b, 25, 29, 30, 31, 32, 34a, 35, 36, 37d, 39, 40, 42, 43, 44, 46, 47, 50, 51
Reclaiming and reorganising the commons	Reclaiming land and property through collective ownership or access (squatting, land recovery)	4, 8, 12, 21, 28, 29, 30, 34, 36, 37b, 47, 51
	Recovering and preserving common resources (water springs, traditional food systems, soil fertility techniques)	5, 16, 23a.b, 33, 35, 37b, 48
	Reorganising collective spaces through sustainable architectural projects	1, 7, 12, 16, 17, 19, 20, 25, 28, 29, 34, 45, 49, 50, 51
Collective deliberative processes	Utilising formal collective organisation structures (assemblies, board meetings, collectives)	3, 4, 7, 8, 12, 14, 20, 21, 22, 25, 27, 28, 30, 32, 34, 35, 37a.b, 40, 41, 43, 46, 47, 48, 51
	Employing informal collaborative processes for planning and/or decision-making (meetings, circles of conversations)	7, 10, 13, 16, 23, 27, 37a.d, 43, 47
	Prioritising consensus-based decision-making and conflict resolution mechanisms	3, 12, 20, 21, 24, 28, 34, 37d, 41, 50, 51
	Formalising protocols and agreements through collective action	3, 4, 8, 14, 21, 30
Political engagement and advocacy	Influencing policy and legislation through collective mobilisation	11, 30, 34b, 35, 37b.c, 38, 49
	Joining anti-globalisation movements	34a, 48, 51
	Lobbying for sustainable construction practices	28a, 34b, 49
	Fighting for environmental causes	10, 34b
	Advocating for animal welfare	18
	Promoting food sovereignty	3, 8, 27, 30, 48
	Advocating for the rights of vulnerable groups	2, 10, 11, 21, 27, 35, 51
	Fighting for land access	2, 4, 8, 27, 48

The first effort (according to point i) is the adoption of alternative forms of production, mainly in agriculture, suggesting a reconnection with the natural cycles of life. The experiences are linked to the principles of agroecology, which may include practices involving seed preservation, reducing or refusing the use of chemical fertilisers and pest control, reducing water use (often through reutilisation), and improve soil regeneration processes, among other actions capable of minimising ecological impacts. This approach extends to forms of consumption and distribution, through selling surpluses or products on local or community markets, hence pointing out to a transformation of the relationship between producers and consumers. The diversification of the logic of production and consumption extends to economic practices, prioritising cooperative-based approaches over profitability and capital accumulation and revealing the adoption of hybrid economic practices.

The second point (item ii), integrating the reorganisation process, relates to reclaiming and reorganising the commons, particularly regarding land and property ownership and other forms of collective access to the living space. This effort to reclaim the commons is also present in the recovery of natural resources or traditional knowledge, as well as in the reorganisation of collective spaces through architectural projects that prioritise the use of environmental-friendly materials and low energy construction methods and solutions.

As regards collective deliberative processes (item iii), the studies revealed resourcing to forms of collective organisation through formal collegiate bodies and informal meetings. Consensus is the primary tool (and objective) to consider in the decision-making process, complemented by mechanisms of conflict resolution. Collective organisations favour the formalisation of protocols or arrangements and changes in policy or legislation.

The organisation of the agents in political groups (item iv), whether formal or informal, is an attribute of some of the initiatives studied (Table 6). Some initiatives express demands linked to historical struggles waged in their regions and by groups marginalised by hegemonic systems (code 2, 3, 4, 5, 8, 13, 14, 21, 23, 27, 29, 30, 35, 37, 38, 42, 46, 48), configuring acts of Resistance. These cases, all located in the Global South, confront colonial and capitalist perspectives, differentiating themselves from other experiences in the sense that the sought transformations are often rooted in the imperative of survival and self-determination, rather than solely on a desire for change. Focusing on agriculture, there is an intention to redeem not only cultural heritage in food production, but also to transform the way “nature is organised” (Felli, 2021, p. 18). This translates to adopting alternative food systems, integrated in a non-extractive model, to provide food sovereignty and autonomy. The approaches of resistance, however, surpass the dynamics of production and consumption and can redefine the socio-ecological relations according to a biocentric logic, comprising education, health, and labour. This approach is intended to reconstruct “social fabric within communities” (Kumbamu 2018, p. 30). Responses to extreme weather events and economic crises can be another catalyst for change. These events go beyond mobilising environmentally conscious individuals (Richardson-Ngwenya 2021) and can foster, in a more pragmatic way, community cohesion, as shown by initiatives in Luxembourg, where residents have been often considered “mainstream and conservative” (Doerr and Taylor Aiken 2021, p. 239).

### Diverse pathways: the influence of territory on transformative approaches

While the previous subsection focused on “change in shape”, a concept etymologically derived from transformation (Hölscher et al. 2018, p. 2), this subsection looked at how different types of initiatives are supported or hindered in their interactions with their territories, drawing on the theoretical framework of transition studies. The development of alternative or radical responses hinges on the diverse experiences and interactions of actors in the territory. These reveal the interplay between material-symbolic practices, existing power relations, and the balance between relational approaches and place-specific characteristics (Miller 1998; Nicolosi and Feola 2016).

In order to understand pathways to socio-ecological changes, it is essential to consider the influence of the territorial context among the different type of initiatives. Peasant-indigenous experiences, for example, often exhibit both intensive and extensive networks, reflecting their deep integration within their communities and their broader socio-political context. This interconnectedness, fostered through economic practices rooted in social and solidarity economies, strengthens their transformative capacity by creating a less dependent support systems from political-institutional elements of the territory. Most of this approach is rooted by regular violence and segregation experienced by their communities, stablishing a necessary network beyond their local net, looking for broader attention-protection and for enhancing collective action. Having a higher degree of integration and interaction contribute to their transformative potential. In contrast, ecovillages primarily rely on intensive relations, which, while fostering strong internal cohesion and social learning, may limit their ability to effect broader societal change. Their focus on self-sufficiency and intentional position in look for alternatives from the hegemonic system can lead to isolation and hinder their capacity to influence wider social and political structures. However, a gradual convergence of practices between IC members’ and their neighbours can also be observed, resulting from mutual influence and growing awareness of social change (e.g. Brombin 2015 and Hong and Vicdan 2016).

This distinction highlights the importance of considering the specific characteristics of each initiative type when assessing their transformative potential. Several factors contribute to or hinder the transformative capacity of these initiatives. Access to resources, including land, capital, and knowledge, plays a crucial role in enabling initiatives to implement their vision and sustain their activities. Strong community support provides legitimacy, resilience, and the social capital necessary for collective action. The political context, including government policies and societal attitudes towards alternative practices, can either enable or constrain the initiatives' ability to operate and scale their impact. Specific practices, such as agroecology, community-based decision-making, and active engagement in political advocacy, appear particularly effective in fostering transformation by promoting social and ecological well-being, empowering communities, and challenging dominant paradigms.

By observing the particularities of spaces, one is allowed to identify the influence material features and natural landscapes have on the actors’ networks and interorganisational relations. In other words, this interconnectedness highlights the importance of “emerging, place-based, and sustainability-oriented experimentations” (Köhler et al. 2019, p. 15). By taking a deeper look at these place-specific approaches, “realistic possibilities for change” towards transformative scenarios can be detected, especially if a “deepening of space-sensitive approaches around scale and site” (Schmid 2019) is used.

### Nuances, vulnerabilities, and implications: the strategies of transformative initiatives

Taking on Feola (2015) and Hölscher et al. (2018), ruptures and discontinuities will be part of any process of change, given its nonlinearity. The cases studied reveal some common vulnerabilities in the context of the dominance of the capitalist system, such as: real estate and land speculation, dispossession, exposition to violent actions, ecosystem degradation, foreign interference that weakens existing social ties, unequal gender relations, and difficulties in shifting away from a (hyper)consumption mindset. However, this context is being challenged by dynamics of trust and actions in search of social protection and self-preservation. This resonates with Polanyi’s concept of countermovement, where communities experiment with ways to address socio-environmental vulnerabilities caused by market expansion (Polanyi 2016 [1944]), exploitation, accumulation and dispossession (Scheidel et al. 2018).

Even considering that each community experiences unique spatial dynamics, with particular characteristics and global connections (Santos 1996), land ownership and use emerge as critical factors. While initiatives from Global North often operate within established legal frameworks that may not fully accommodate their collective land ownership models (sometimes even employing religious classification to make viable a collective ownership), initiatives from Global South frequently face challenges related to land access and tenure security. Furthermore, the economic systems available to these groups significantly influence their capacity to challenge capitalist logic. Peasant-indigenous movements, often rooted in social and solidarity economies, may have greater potential to create alternative economic models compared to ecovillages, which may still rely on market-based interactions and on social capital from their previous lifestyle. A deeper understanding of these economic distinctions is crucial for accurately assessing the transformative potential of different initiatives and ensuring a consistent application of instruments for changes.

Although often evaluated through an economic lens, the transformative potential of the initiatives extends beyond mere economic viability. While economic sustainability is undeniably crucial, particularly for initiatives striving for self-sufficiency, it is essential to recognise the broader economic dimensions at play. Transformative initiatives frequently embody relational economies and practices of reciprocity. Assessing their effectiveness requires considering non-monetary aspects like social cohesion, ecological well-being, and cultural values. Furthermore, the pursuit of alternative economic models often necessitates challenging existing legal and institutional frameworks that prioritise capitalist principles of property and resource allocation (Leff 2001).

Addressing these challenges requires socio-environmental policies grounded in real-world experiences (Castree 2002; Robbins 2003). Policy recommendations should consider the specific needs and contexts of different initiative types. For example, rethinking land use regulations and zoning policies to accommodate eco-centric practices can benefit both northern and southern initiatives. Ultimately, it can stimulate ideas and proposals for a collective model of sustainability. However, it also requires reconsidering the demand for natural resources from North Global inhabitants and addressing power relations to tackle the roots of vulnerabilities. The initiatives, as exemplified by the 88 cases studied, have a crucial educational role to play, advocating for policy changes that recognise the value of alternative economic models and promote a more just and sustainable ways of life.

## Conclusion

This study contributes to the development of the Transformative Initiative concept by identify alternative practices that enhance social and environmental justice through less resource-intensive and more self-sufficient ways of living. The analysis focused on identifying core principles, dimensions, patterns, and practices employed within transformative initiatives studied by academic research.

The results reveal a significant difference between the experiences taking place in the Global North and in the Global South. The changes in the Global North tend to align with degrowth proposals or intentional communities like ecovillages, while the experiences in the Global South tend to emerge from movements or actions of resistance (when not survival) linked to post-development and other alternative ontologies (Escobar 2015; Garcia-Arias and Schöneberg 2021). These experiences evolving in both regions owe a lot to its marginal ontological location regarding the major Global political and economic power hubs. This marginalisation compels people and communities to develop these initiatives out of either inspiration or desperation (Shields 1991; Radcliffe 2015).

These experiences reveal a tendency to reshape the relationship between individuals as well as between humans and non-human entities. Technological advances have been used as auxiliary tools to facilitate the process of reconnecting people with the *milieu*. The ScR demonstrates that intentional communities and peasant and indigenous movements (78% of the initiatives in the study are of both types) tend to seek alternatives to production and consumption through agroecology, preservation of ancestral practices and reforestation actions. This (re)connection with biophysical rhythms was not limited to rural environments, as it was also observed in urban experiences, in this case through time management initiatives and through the adoption of building practices involving less construction and maintenance impacts. Initiatives such as community gardens or agricultural practices based on permaculture, reinstating the connection with the land into their routines, were also observed. Fostering dialogue around these evolving practices and values, and potentially institutionalising them, is viewed as another avenue for strengthening social transformation.

In the process of “social learning” (Few et al. 2017, p. 4), the agents establish “new communal political subjects” (Valencia and Courtheyn 2023, p. 1093). By doing so, the agents diversify economic logics, create different understandings of land ownership, of the relation between time, labour, and capital, incorporate alternative cosmologies and restore the joy of social relations. These changes occur at the local level, where local and global interactions enable a deeper understanding of the world as it currently exists or may evolve in the future. This is part of a continuous process of transforming boundaries and shared practices (Santos 1996)

The ScR findings provide valuable insights for policymakers seeking to foster transformative initiatives, highlighting the importance of supporting diverse pathways to sustainability, recognising the unique challenges faced by different types of initiatives in different contexts, and enabling environments that promote social and ecological well-being.

The cases studied unfold a multitude of alternative approaches from various regions around the world. As Ulug et al. (2021, p. 15) argue, “recognising marginal projects could reveal their potential as fertile ground for experimental practices and identify gaps in our unsustainable society”. This thought can illuminate the construction of the Pluriverse (Kothari et al. 2019), a concept that acknowledges the right for different possible worlds to coexist (Leff 2001).

## Supplementary Information

Below is the link to the electronic supplementary material.

Supplementary file1 (PDF 291 KB)

## Footnotes

1 Escobar (2015) explains that he opted for the term 'transition' instead of ‘transformation’ because the former was more commonly used within the frameworks that he analysed.

2 Each stage (title, abstract and full-text) has followed the same screening process, using inclusion and exclusion criteria adapted from Societal Transformation study (Feola 2015). The same inclusion and exclusion process overlaps the whole study, each with a different depth of analyses: from the original analyses of the data to the body of variables composing the final selection of documents.

## References

[CR1] Acosta, A. 2016. *O bem viver: uma oportunidade para imaginar outros mundos* (Ed.). https://rosaluxspba.org/wp-content/uploads/2017/06/Bemviver.pdf.

[CR2] Alimonda, H. (Ed.). 2011. *La Naturaleza colonizada. Ecología política y minería en América Latina*. CLACSO. https://biblioteca.clacso.edu.ar/clacso/gt/20120319035504/natura.pdf.

[CR3] Althor, G., and B. Witt. 2020. A quantitative systematic review of distributive environmental justice literature: A rich history and the need for an enterprising future. *Journal of Environmental Studies and Sciences* 10: 91–103. 10.1007/s13412-019-00582-9.

[CR4] Arksey, H., and L. O’Malley. 2005. Scoping studies: Towards a methodological framework. *International Journal of Social Research Methodology: Theory and Practice* 8: 19–32. 10.1080/1364557032000119616.

[CR5] Atutxa, E., I. Zubero, and I. Calvo-Sotomayor. 2020. Scalability of low carbon energy communities in spain: An empiric approach from the renewed commons paradigm. *Energies* 13: 5045. 10.3390/en13195045.

[CR6] Benjaminsen, T.A., and H. Svarstad. 2018. Political ecology. In *Encyclopedia of ecology*, ed. B. Fath, 391–396. Berlin: Elsevier Ltd.

[CR7] Beraldo, D., and S. Milan. 2019. From data politics to the contentious politics of data. *Big Data and Society* 6: 2053951719885967. 10.1177/2053951719885967.

[CR201] Brombin, A. 2015. Faces of sustainability in Italian ecovillages: Food as ‘contact zone’. *International Journal of Consumer Studies* 39: 468–477. 10.1111/ijcs.12225.

[CR8] Burke, B.J., and B. Shear. 2014. Introduction: Engaged scholarship for non-capitalist political ecologies. *Journal of Political Ecology* 21: 127–144. 10.2458/v21i1.21128.

[CR9] Castree, N. 2002. Environmental issues: From policy to political economy. *Progress in Human Geography* 26: 357–366. 10.1191/0309132502ph374pr.

[CR10] Cavanagh, C.J., and T.A. Benjaminsen. 2017. Political ecology, variegated green economies, and the foreclosure of alternative sustainabilities. *Journal of Political Ecology* 24: 200–216. 10.2458/v24i1.20800.

[CR11] Dahle, K. 2007. When do transformative initiatives really transform? A typology of different paths for transition to a sustainable society. *Futures* 39: 487–504. 10.1016/j.futures.2006.10.007.

[CR12] Danowski, D., and E. Viveiros de Castro. 2017. *The ends of the world (Translated by Rodrigo Nunes)*. English. Polity Press.

[CR13] Doerr, J.T., and G. Taylor Aiken. 2021. Transformative pragmatism: How a diversity of Leitbilder is harnessed for rural transformation in Réiden, Luxembourg. *Environmental Policy and Governance* 31: 237–248. 10.1002/eet.1932.

[CR14] Escobar, A. 2015. Degrowth, postdevelopment, and transitions: a preliminary conversation. *Sustainability Science* 10: 451–462. 10.1007/s11625-015-0297-5.

[CR15] Fèche, R., C. Noûs, and F. Barataud. 2021. Building a transformative initiative for a territorialized agri-food system: constructing a living-lab and confronting norms? A case study from Mirecourt (Vosges, France). *Journal of Rural Studies* 88: 400–409. 10.1016/j.jrurstud.2021.07.026.

[CR200] Felli, R. 2021. *The great adaptation: Climate, capitalism and catastrophe*. Translated by David Broder. Verso.

[CR16] Feola, G. 2015. Societal transformation in response to global environmental change: A review of emerging concepts. *Ambio* 44: 376–390. 10.1007/s13280-014-0582-z. 25431335 10.1007/s13280-014-0582-z PMC4510318

[CR17] Feola, G. 2020. Capitalism in sustainability transitions research: Time for a critical turn? *Environmental Innovation and Societal Transitions* 35: 241–250. 10.1016/j.eist.2019.02.005.

[CR18] Feola, G., and S. Jaworska. 2018. One transition, many transitions? A corpus-based study of societal sustainability transition discourses in four civil society’s proposals. *Sustainability Science* 14: 1643–1656. 10.1007/s11625-018-0631-9.

[CR19] Few, R., D. Morchain, D. Spear, A. Mensah, and R. Bendapudi. 2017. Transformation, adaptation and development: Relating concepts to practice. *Palgrave Communications* 3: 1–9. 10.1057/palcomms.2017.92.

[CR20] Galviz, C.L., and E. Spiers, eds. 2022. *Routledge handbook of social futures*. Routledge.

[CR21] Garcia-Arias, J., and J. Schöneberg. 2021. Urgencies and imperatives for revolutionary (environmental) transitions: From degrowth and postdevelopment towards the pluriverse? *Environmental Politics* 30: 865–871. 10.1080/09644016.2021.1911443.

[CR22] Geels, F.W. 2011. The multi-level perspective on sustainability transitions: Responses to seven criticisms. *Environmental Innovation and Societal Transitions* 1: 24–40. 10.1016/j.eist.2011.02.002.

[CR23] Gorissen, L., F. Spira, E. Meynaerts, P. Valkering, and N. Frantzeskaki. 2018. Moving towards systemic change? Investigating acceleration dynamics of urban sustainability transitions in the Belgian City of Genk. *Journal of Cleaner Production* 173: 171–185. 10.1016/j.jclepro.2016.12.052.

[CR24] Hans de Haan, J., and J. Rotmans. 2011. Patterns in transitions: Understanding complex chains of change. *Technological Forecasting and Social Change* 78: 90–102. 10.1016/j.techfore.2010.10.008.

[CR25] Hansen, T., and L. Coenen. 2015. The geography of sustainability transitions: Review, synthesis and reflections on an emergent research field. *Environmental Innovation and Societal Transitions* 17: 92–109. 10.1016/j.eist.2014.11.001.

[CR26] Hölscher, K., J.M. Wittmayer, and D. Loorbach. 2018. Transition versus transformation: What’s the difference? *Environmental Innovation and Societal Transitions* 27: 1–3. 10.1016/j.eist.2017.10.007.

[CR202] Hong, S., and H. Vicdan. 2016. Re-imagining the utopian: Transformation of a sustainable lifestyle in ecovillages. *Journal of Business Research* 69: 120–136. 10.1016/j.jbusres.2015.07.026.

[CR27] Köhler, J., F.W. Geels, F. Kern, J. Markard, E. Onsongo, A. Wieczorek, F. Alkemade, F. Avelino, et al. 2019. An agenda for sustainability transitions research: State of the art and future directions. *Environmental Innovation and Societal Transitions* 31: 1–32. 10.1016/j.eist.2019.01.004.

[CR28] Kothari, A., A. Salleh, A. Escobar, F. Demaria, and A. Acosta, eds. 2019. *Pluriverse: A post-development dictionary*. Tulika Books.

[CR29] Kumbamu, A. 2018. Building sustainable social and solidarity economies: Place-based and network-based strategies of alternative development organizations in India. *Community Development* 49: 18–33. 10.1080/15575330.2017.1384744.

[CR31] Leff, E. 2001. *Saber ambiental: Sustentabilidade, racionalidade, complexidade, poder*. Vozes.

[CR32] Longhurst, N. 2013. The emergence of an alternative milieu: Conceptualising the nature of alternative places. *Environment and Planning A* 45: 2100–2119. 10.1068/a45487.

[CR33] Loorbach, D., N. Frantzeskaki, and F. Avelino. 2017. Sustainability transitions research: Transforming science and practice for societal change. *Annual Review of Environment and Resources* 42: 599–626. 10.1146/annurev-environ-102014-021340.

[CR34] Miller, B. 1998. Justice, nature, and the geography of difference. *David Harvey. Urban Geography* 19: 777–781. 10.2747/0272-3638.19.8.777.

[CR35] Nicolosi, E., and G. Feola. 2016. Transition in place: Dynamics, possibilities, and constraints. *Geoforum* 76: 153–163. 10.1016/j.geoforum.2016.09.017.

[CR36] Page, M.J., J.E. McKenzie, P.M. Bossuyt, I. Boutron, T.C. Hoffmann, C.D. Mulrow, L. Shamseer, J.M. Tetzlaff, et al. 2021. The PRISMA 2020 statement: An updated guideline for reporting systematic reviews. *Systematic Reviews* 10: n160. 10.1186/s13643-021-01626-4. 10.1186/s13643-021-01626-4 PMC8008539 33781348

[CR37] Peet, R., W.P. Robbins, and T.M. Watts. 2010. Global nature. In *Global political ecology*, ed. R. Peet, W.P. Robbins, and T.M. Watts, 1–48. Routledge.

[CR38] Pereira, L.M., T. Karpouzoglou, N. Frantzeskaki, and P. Olsson. 2018. Designing transformative spaces for sustainability in social-ecological systems. *Ecology and Society* 23: 32. 10.5751/ES-10607-230432.

[CR204] Polanyi, K. (2016 [1944]). A grande transformação. Edições 70

[CR39] Radcliffe, S.A. 2015. Development alternatives. *Development and Change* 46: 855–874. 10.1111/dech.12179.

[CR40] Raudsepp-Hearne, C., G.D. Peterson, E.M. Bennett, R. Biggs, A.V. Norström, L. Pereira, J. Vervoort, D.M. Iwaniec, et al. 2020. Seeds of good anthropocenes: Developing sustainability scenarios for Northern Europe. *Sustainability Science* 15: 605–617. 10.1007/s11625-019-00714-8.

[CR41] Richardson-Ngwenya, P. 2021. Everyday political geographies of community-building: Exploring the practices of three Zimbabwean permaculture communities. *Environmental Policy and Governance* 31: 211–222. 10.1002/eet.1930.

[CR42] Robbins, P. 2003. Political ecology in political geography. *Political Geography* 22: 641–645. 10.1016/S0962-6298(03)00071-4.

[CR43] Rodriguez-Labajos, B. 2022. Artistic activism promotes three major forms of sustainability transformation. *Current Opinion in Environmental Sustainability* 57: 101199. 10.1016/j.cosust.2022.101199.

[CR44] Ross, A., and R. Jones. 2016. Connections and tensions between nationalist and sustainability discourses in the Scottish Legislative Process. *Journal of Law and Society* 43: 228–256. 10.1111/j.1467-6478.2016.00750.x.

[CR45] Roysen, R., and T.C. Cruz. 2020. Educating for transitions: ecovillages as transdisciplinary sustainability “classrooms.” *International Journal of Sustainability in Higher Education* 21: 977–992. 10.1108/IJSHE-01-2020-0009.

[CR46] Santos, M. 1996. O lugar: Encontrando o futuro. *Revista De Urbanismo e Arquitetura* 4: 35–39.

[CR47] Scheidel, A., L. Temper, F. Demaria, and J. Martinez-Alier. 2018a. Ecological distribution conflicts as forces for sustainability: An overview and conceptual framework. *Sustainability Science* 13: 585–598. 10.1007/s11625-017-0519-0. 30147788 10.1007/s11625-017-0519-0 PMC6086280

[CR49] Schlosberg, D. 2007. *Defining environmental justice: Theories, movements, and nature*, vol. 9780199286. Berlin: Oxford University Press.

[CR50] Schmid, B. 2019. Degrowth and postcapitalism: Transformative geographies beyond accumulation and growth. *Geography Compass* 13: e12470. 10.1111/gec3.12470.

[CR51] Schmid, B., and T.S.J. Smith. 2021. Social transformation and postcapitalist possibility: Emerging dialogues between practice theory and diverse economies. *Progress in Human Geography* 45: 253–275. 10.1177/0309132520905642.

[CR52] Schot, J., and F.W. Geels. 2008. Strategic niche management and sustainable innovation journeys: Theory, findings, research agenda, and policy. *Technology Analysis and Strategic Management* 20: 537–554. 10.1080/09537320802292651.

[CR53] Shields, R. 1991. Synthesis and implications in *Places on the margin: Alternative geographies of modernity*. Routledge Taylor & Francis Group. 10.2307/2580500.

[CR54] Sovacool, B.K., and D.J. Hess. 2017. Ordering theories: Typologies and conceptual frameworks for sociotechnical change. *Social Studies of Science* 47: 703–750. 10.1177/0306312717709363. 28641502 10.1177/0306312717709363 PMC5648049

[CR55] Tricco, A.C., E. Lillie, W. Zarin, K.K. O’Brien, H. Colquhoun, D. Levac, D. Moher, M.D.J. Peters, et al. 2018. PRISMA extension for scoping reviews (PRISMA-ScR): Checklist and explanation. *Annals of Internal Medicine* 169: 467–473. 10.7326/M18-0850. 30178033 10.7326/M18-0850

[CR56] Ulug, C., L. Horlings, and E.-M. Trell. 2021. Collective identity supporting sustainability transformations in ecovillage communities. *Sustainability (Switzerland)* 13: 8148. 10.3390/su13158148.

[CR57] Unanue, I., S.G. Patel, T.T. Tormala, C.D. Trott, A.A. Piazza Rodríguez, K.M. Serrano, and L.M. Brown. 2020. Seeing more clearly: Communities transforming towards justice in post-hurricane Puerto Rico. *Community Psychology in Global Perspective* 6: 22–47.

[CR58] Valencia, Ó.E., and C. Courtheyn. 2023. Peace through coca? Decolonial peacebuilding ecologies and rural development in the Territory of Conviviality and Peace of Lerma, Colombia. *Third World Quarterly* 44: 1077–1097. 10.1080/01436597.2023.2175656.

[CR59] Wolfram, M. 2016. Conceptualizing urban transformative capacity: A framework for research and policy. *Cities* 51: 121–130. 10.1016/j.cities.2015.11.011.

[CR60] Wolfram, M., S. Borgström, and M. Farrelly. 2019. Urban transformative capacity: From concept to practice. *Ambio* 48: 437–448. 10.1007/s13280-019-01169-y. 30903513 10.1007/s13280-019-01169-y PMC6462303

