# Supplementary material for: Mapping, characterisation, and analysis of initiatives with transformative capacity: A scoping review
Source: Ambio. 2025 Feb 14;54(6):947–64. doi: 10.1007/s13280-025-02133-9 (PMC12055674; doi:10.1007/s13280-025-02133-9)
Supplement: Supplementary file 1 — Supplementary file1 (PDF 291 KB) [file 13280_2025_2133_MOESM1_ESM.pdf]

Ambio

Supplementary Information
This supplementary information has not been peer reviewed.

Title: **Mapping, characterisation, and analysis of initiatives with transformative capacity: A scoping review**

Characteristics and results of each case studied (n=88**) by publication (**n=51**)**: In the following table, the characteristics of each study are presented and charted in order to meet the central question and the objectives of the ScR: "What type of initiatives with transformative capacity, focused on a socioecological approach, can be identified and analysed in scientific indexed databases?".

Code[1]	Publication	Country	Type of Initiative	Starting year	Mechanism of change	Territory Relations	Transformative Space[2]
**1**	(Pisters et al., 2023)	Finland	Ecovillage, village	n.i.[3]	Reorientation	Intensive and Extensive	1.2.4.
**1**	(Pisters et al., 2023)	Finland	Ecovillage, village	1978	Reorientation	Intensive and Extensive	1.2.4.
**1**	(Pisters et al., 2023)	Portugal	Ecovillage, village	1995	Reorientation	Intensive and Extensive	1.2.4.

[1] *Codes refer to the publication following the title alphabetically, some containing more than one case study.*
[2] *Territory space characteristics (based on Pereira et al., 2018): 1. recognition of need to change the dominant system; 2. presence of mobilizing agents; 3. ability to integrate diverse actors; 4. readiness to seize opportunities; 5. Fusing diverse worldviews as catalyst of transformative changes.*
[3] *n.i. = not informed or not identified.*

**2**	(Feola et al., 2021)	Colombia	Peasant-Indigenous movements, settlements	2016	Innovation; 2 Re..[4]; Resistance	Intensive and Extensive	1.2.3.4.5.
**3**	(Jimenez et al., 2022)	Peru	Peasant-Indigenous movements, settlements	2000	Innovation; 2 Re..; Resistance	Intensive and Extensive	1.2.3.4.5.
**4**	(Forbis, 2016)	Mexico	Peasant-Indigenous movements, settlements	1994	2 Re..; Resistance	Intensive and Extensive	1.2.3.4.
**5**	(Levidow et al., 2021)	Brazil	Peasant-Indigenous movements, settlements	n.i.	2 Re..; Resistance	Intensive and Extensive	1.2.3.4.5.
**5**	(Levidow et al., 2021)	Brazil	Peasant-Indigenous movements, settlements	2003	2 Re..; Resistance	Intensive and Extensive	1.2.3.4.5.
**6**	(Wilson, 2013)	USA	Cooperatives, Associations	n.i.	Reorganisation	Intensive	1.4.
**6**	(Wilson, 2013)	USA	Project	1970s	Reorganisation	Intensive	1.4.
**7**	(Tummers & MacGregor, 2019)	Netherlands	Urban, peri-urban collective	1980s	Reorganisation	Intensive	1.2.
**7**	(Tummers & MacGregor, 2019)	Netherlands	Urban, peri-urban collective	1980s	Reorganisation	Intensive	1.2.
**7**	(Tummers & MacGregor, 2019)	UK	Urban, peri-urban collective	1980s	Reorganisation	Intensive	1.2.
**7**	(Tummers & MacGregor, 2019)	UK	Urban, peri-urban collective	1980s	Reorganisation	Intensive	1.2.

[4] *2Re.. = **Re**orientation and **Re**organisation*

**8**	(Gahman, 2017)	Mexico	Peasant-Indigenous movements, settlements	1994	2 Re..; Resistance	Intensive and Extensive	1.2.3.4.
**9**	(Kumbamu, 2018)	India	Cooperatives, Associations	2004	2 Re..	Intensive and Extensive	1.2.3.4.5.
**10**	(Kingfisher, 2022)	Canada	Urban, peri-urban collective	1998	2 Re..	Intensive and Extensive	1.2.
**10**	(Kingfisher, 2022)	Japan	Urban, peri-urban collective	2003	2 Re..	Intensive	1.2.
**11**	(Ulug et al., 2021)	USA	Ecovillage, village	1967	2 Re..	intensive	1.2.4.
**11**	(Ulug et al., 2021)	USA	Ecovillage, village	1989	2 Re..	intensive	1.2.4.
**11**	(Ulug et al., 2021)	USA	Ecovillage, village	1993	2 Re..	Intensive and Extensive	1.2.4.
**12**	(Lockyer, 2017)	USA	Ecovillage, village	1990s	2 Re..	Intensive	1.2.4.
**13**	(Burke & Arjona, 2013)	Colombia	Ecovillage, village	2003	2 Re..; Resistance	Intensive and Extensive	1.2.4.
**14**	(Nirmal & Rocheleau, 2019)	Mexico	Peasant-Indigenous movements, settlements	1980s	2 Re..; Resistance	Intensive and Extensive	1.2.4.
**15**	(Rocha, 2022)	Brazil	Project	n.i.	Reorientation	Intensive	1.2.
**16**	(Muñoz-Villarreal, 2018)	Colombia	Ecovillage, village	2006	2 Re..	Intensive	1.2.3.4.
**16**	(Muñoz-Villarreal, 2018)	Colombia	Ecovillage, village	2006	2 Re..	Intensive	1.2.3.4.
**17**	(Flores & Trevizan, 2017)	Brazil	Ecovillage, village	2002	Innovation; Reorganisation	Intensive	1.4.

**18**	(Moravcikova & Furjeszova, 2018)	Hungary	Ecovillage, village	1993	2 Re..	Intensive and Extensive	1.2.4.
**18**	(Moravcikova & Furjeszova, 2018)	Hungary	Ecovillage, village	1996	2 Re..	Intensive and Extensive	1.2.4.
**18**	(Moravcikova & Furjeszova, 2018)	Slovakia	Ecovillage, village	1990s	2 Re..	Intensive and Extensive	1.2.4.
**19**	(Rubin, 2019)	USA	Ecovillage, village	n.i.	Innovation; 2 Re..	Intensive and Extensive	1.2.4.5.
**20**	(Ergas & Clement, 2016)	USA	Ecovillage, village	n.i.	2 Re..	Intensive	1.2.
**21**	(Luna, 2013)	Mexico	Peasant-Indigenous movements, settlements	1983	2 Re..; Resistance	Intensive and Extensive	1.2.3.4.
**22**	(Fois, 2019)	Italy	Ecovillage, village	1979	2 Re..	Intensive	2.3.4.
**23**(a)	(Richardson-Ngwenya, 2021)	Zimbabwe	Project	1991	2 Re..; Resistance	Intensive	1.2.3.4.
**23**(b)	(Richardson-Ngwenya, 2021)	Zimbabwe	Ecovillage, village	2003	2 Re..; Resistance	Intensive	1.2.3.4.
**23**(c)	(Richardson-Ngwenya, 2021)	Zimbabwe	Project	2006	2 Re..; Resistance	Intensive	1.2.3.4.
**24**(a)	(Brombin, 2015)	Italy	Ecovillage, village	1979	2 Re..	Intensive	1.2.4.
**24**(b)	(Brombin, 2015)	Italy	Ecovillage, village	1995	2 Re..	Intensive	1.2.4.
**24**(c)	(Brombin, 2015)	Italy	Ecovillage, village	2011	2 Re..	Intensive	1.2.4.
**25**	(Price et al., 2020)	Australia	Ecovillage, village	2013	Reorientation	Intensive	1.2.4.

**26**	(Moreira & Fuster Morell, 2020)	Portugal	Cooperatives, Associations	2014	Innovation; Reorganisation	Intensive	1.2.4.
**27**	(De Mari et al., 2017)	Brazil	Peasant-Indigenous movements, settlements	1990s	2 Re..; Resistance	Intensive and Extensive	1.2.3.4.5.
**28**	(Boyer, 2015)	USA	Ecovillage, village	1992	2 Re..	Intensive and Extensive	1.2.4.
**28**	(Boyer, 2015)	USA	Ecovillage, village	1993	2 Re..	Intensive	1.2.3.4.5.
**28**	(Boyer, 2015)	USA	Ecovillage, village	1997	2 Re..	Intensive	1.2.4.
**29**	(Morris, 2022)	Mexico	Ecovillage, village	n.i.	2 Re..; Resistance	Intensive and Extensive	1.2.4.
**29**	(Morris, 2022)	Mexico	Ecovillage, village	2015	2 Re..; Resistance	Intensive and Extensive	1.2.4.
**30**	(Fernandes et al., 2021)	Brazil	Peasant-Indigenous movements, settlements	1986	2 Re..; Resistance	Intensive and Extensive	1.2.4.5.
**31**	(Gorissen et al., 2018)	Belgium	Project	n.i.	2 Re..	Intensive and Extensive	2.3.4.5.
**32**	(Lloveras et al., 2020)	Spain	Alternative currency networking	2011	2 Re..	Intensive	1.2.4.
**33**	(Javier & Sastoque, 2012)	Colombia	Ecovillage, village	n.i.	2 Re..	Intensive	1.2.4.
**34**(a)	(Nelson, 2018)	USA	Ecovillage, village	1967	2 Re..	Intensive and Extensive	1.2.4.
**34**(b)	(Nelson, 2018)	Australia	Urban, peri-urban collective	1971	2 Re..	Intensive	1.2.4.
**35**	(Valencia & Courtheyn, 2023)	Colombia	Peasant-Indigenous movements, settlements	2013	2 Re..; Resistance	Intensive and Extensive	1.2.3.4.5.
**36**	(Bendix et al., 2019)	Germany	Cooperatives, Associations	1990s	Innovation; Reorganisation	Intensive	1.2.

**37**(a)	(Schöneberg, 2019)	Haiti	Peasant-Indigenous movements, settlements	2001	2 Re..; Resistance	Intensive and Extensive	2.3.
**37**(b)	(Lang, 2019)	Ecuador	Peasant-Indigenous movements, settlements	2007	Innovation; 2 Re..; Resistance	Intensive and Extensive	1.2.3.4.5.
**37**(c)	(Caria & Domínguez, 2019)	Ecuador	Peasant-Indigenous movements, settlements	2008	Innovation; 2 Re..	Intensive	1.2.3.4.5.
**37**(d)	(Chitranshi, 2019)	India	Ecovillage, village	2016	2 Re..; Resistance	Intensive	1.4.
**38**	(Coombe & Jefferson, 2021)	Colombia	Peasant-Indigenous movements, settlements	1991	Innovation; 2 Re..; Resistance	Intensive and Extensive	1.2.3.4.5.
**38**	(Coombe & Jefferson, 2021)	Ecuador	Peasant-Indigenous movements, settlements	2008	Innovation; 2 Re..; Resistance	Intensive and Extensive	1.2.3.4.5.
**39**	(Macías-Macías & Sevilla-García, 2020)	Mexico	Peasant-Indigenous movements, settlements	2013	2 Re..	Intensive	1.2.3.4.
**40**	(Chaves et al., 2018)	Colombia	Peasant-Indigenous movements, settlements	2012	2 Re..	intensive	1.2.3.4.
**41**	(Hong & Vicdan, 2016)	USA	Ecovillage, village	1990s	2 Re..	Intensive	1.2.4.
**42**	(North, 2005)	Argentina	Alternative currency networking	1997	Expansion; Reorganisation; Resistance	Intensive and Extensive	1.2.4.
**42**	(North, 2005)	UK	Alternative currency networking	1992	Expansion; Reorganisation	Intensive	1.2.4.
**43**	(Grasseni, 2014)	Italy	Cooperatives, Associations	1994	2 Re..	Intensive	1.2.3.4.

**44**	(Chitranshi & Healy, 2022)	Australia	Cooperatives, Associations	1895	Reorganisation	intensive	1.2.3.4.
**44**	(Chitranshi & Healy, 2022)	India	Cooperatives, Associations	2014	2 Re..	intensive	1.2.4.
**45**	(Borsos, 2009)	Hungary	Ecovillage, village	1991	Innovation; Reorganisation	n.i.	1.2.4.
**46**	(Uharte, 2022)	Venezuela	Urban, peri-urban collective	2008	Reorganisation; Resistance	Intensive and Extensive	1.2.3.4.
**47**	(Cattaneo & Gavaldà, 2010)	Spain	Urban, peri-urban collective	1996	Innovation; 2 Re..	Intensive and Extensive	1.2.4.
**47**	(Cattaneo & Gavaldà, 2010)	Spain	Urban, peri-urban collective	2001	Innovation; 2 Re..	Intensive and Extensive	1.2.4.
**48**	(Schöneberg et al., 2022)	Tanzania	Peasant-Indigenous movements, settlements	n.i.	2 Re..; Resistance	Intensive	1.2.4.
**48**	(Schöneberg et al., 2022)	Haiti	Peasant-Indigenous movements, settlements	n.i.	2 Re..; Resistance	Intensive and Extensive	1.2.4.
**49**	(Doerr & Taylor Aiken, 2021)	Luxembourg	Project	1998	2 Re..	Intensive	1.2.3.4.5.
**49**	(Doerr & Taylor Aiken, 2021)	Luxembourg	Project	2001	2 Re..	Intensive	1.2.3.4.5.
**49**	(Doerr & Taylor Aiken, 2021)	Luxembourg	Alternative currency networking	2013	2 Re..	Intensive	1.2.3.4.5.
**49**	(Doerr & Taylor Aiken, 2021)	Luxembourg	Cooperatives, Associations	2016	2 Re..	Intensive	1.2.3.4.5.
**50**	(Casey et al., 2017)	Ireland	Ecovillage, village	n.i.	Innovation; 2 Re..	Intensive	1.2.3.4.
**51**	(Miles, 2008)	Denmark	Urban, peri-urban collective	1971	Innovation; 2 Re..	Intensive and Extensive	1.2.4.
**51**	(Miles, 2008)	Germany	Urban, peri-urban collective	1979	2 Re..	Intensive and Extensive	1.2.4.

**51**	(Miles, 2008)	Germany	Ecovillage, village	1991	Innovation; 2 Re..	Intensive and Extensive	1.2.3.4.5.
**51**	(Miles, 2008)	USA	Urban, peri-urban collective	1996	2 Re..	Intensive	2.4.
**51**	(Miles, 2008)	USA	Ecovillage, village	1996	Innovation; 2 Re..	Intensive	2.4.
**51**	(Miles, 2008)	India	Ecovillage, village	1960s	Innovation; 2 Re..	Intensive and Extensive	1.2.3.4.5.

Reference

Bendix, D., Müller, F., & Ziai, A. (2019). Postdevelopment alternatives in the north. In *Postdevelopment in Practice: Alternatives, Economies, Ontologies*. https://www.scopus.com/inward/record.uri?eid=2-s2.0-85128051460&partnerID=40&md5=3f5bedb7e03b947c638cc747559475ab

Borsos, B. (2009). Systems theory and ecological settlement design: A pilot project in rural Hungary. *Hungarian Studies*, *23*(2), 175–194. https://doi.org/10.1556/HStud.23.2009.2.2

Boyer, R. H. W. (2015). Grassroots innovation for urban sustainability: Comparing the diffusion pathways of three ecovillage projects. *Environment and Planning A*, *47*(2), 320–337. https://doi.org/10.1068/a140250p

Brombin, A. (2015). Faces of sustainability in Italian ecovillages: Food as "contact zone." *International Journal of Consumer Studies*, *39*(5), 468–477. https://doi.org/10.1111/ijcs.12225

Burke, B. J., & Arjona, B. (2013). Creating alternative political ecologies through the construction of ecovillages and ecovillagers in Colombia. In *Environmental Anthropology Engaging Ecotopia: Bioregionalism, Permaculture, and Ecovillages* (Vol. 17). https://www.scopus.com/inward/record.uri?eid=2-s2.0-84917470240&partnerID=40&md5=0f189366558c04cd8ba428710aeb465e

Caria, S., & Domínguez, R. (2019). Postdevelopment's forgotten 'other roots' in the Spanish and Latin American history of development thought. In Klein, E., & Morreo, C. E. (Eds.). *Postdevelopment in practice: Alternatives, economies, ontologies* (pp.52-65). https://doi.org/10.4324/9780429492136

Casey, K., Lichrou, M., & O'Malley, L. (2017). Unveiling Everyday Reflexivity Tactics in a Sustainable Community. *Journal of*

Macromarketing, *37*(3), 227–239. https://doi.org/10.1177/0276146716674051
Cattaneo, C., & Gavaldà, M. (2010). The experience of rurban squats in Collserola, Barcelona: what kind of degrowth? *Journal of Cleaner Production*, *18*(6), 581–589. https://doi.org/10.1016/j.jclepro.2010.01.010
Chaves, M., Macintyre, T., Verschoor, G., & Wals, A. E. J. (2018). Radical ruralities in practice: Negotiating buen vivir in a Colombian network of sustainability. *Journal of Rural Studies*, *59*, 153–162. https://doi.org/10.1016/j.jrurstud.2017.02.007
Chitranshi, B. (2019). Beyond development: postcapitalist and feminist praxis in adivasi contexts. In Klein, E., & Morreo, C. E. (Eds.). *Postdevelopment in practice: Alternatives, economies, ontologies* (pp.119-132). https://doi.org/10.4324/9780429492136
Chitranshi, B., & Healy, S. (2022). Shared survival and cooperation in India and Australia. *Asia Pacific Viewpoint*, *63*(1), 151–162. https://doi.org/10.1111/apv.12335
Coombe, R. J., & Jefferson, D. J. (2021). Posthuman rights struggles and environmentalisms from below in the political ontologies of Ecuador and Colombia. *Journal of Human Rights and the Environment*, *12*(2), 177–204. https://doi.org/10.4337/jhre.2021.02.02
De Mari, C. L., Villas Boas Tavares, P. D., & da Fonseca, V. M. (2017). Food, knowledge and education for the "good living": the campones a step ahead. *REMEA-Revista Eletrônica Do Mestrado Em Educação Ambiental*, *34*(3), 37–54. https://doi.org/10.14295/remea.v34i3.7181
Doerr, J. T., & Taylor Aiken, G. (2021). Transformative pragmatism: How a diversity of Leitbilder is harnessed for rural transformation in Réiden, Luxembourg. *Environmental Policy and Governance*, *31*(3), 237–248. https://doi.org/10.1002/eet.1932
Ergas, C., & Clement, M. T. (2016). Ecovillages, Restitution, and the Political-Economic Opportunity Structure: An Urban Case Study in Mitigating the Metabolic Rift. *Critical Sociology*, *42*(7–8), 1195–1211. https://doi.org/10.1177/0896920515569085
Feola, G., Vincent, O., & Moore, D. (2021). (Un)making in sustainability transformation beyond capitalism. *Global Environmental Change*, *69*. https://doi.org/10.1016/j.gloenvcha.2021.102290
Fernandes, I. F., Barbosa, L. P., Dos Santos Damasceno, C., & Rosset, P. M. (2021). Inventory of Agroecological Practices as part of the "Peasant to Peasant" Methodology in Ceará: An instrument to decolonize a territory and (re)value peasant knowledge [Inventário de Práticas Agroecológicas na Metodologia "de Camponês/a a Camponês/a" no C. *Desenvolvimento e Meio Ambiente*, *58*, 551–578. https://doi.org/10.5380/dma.v58i0.77777
Flores, B. N., & Trevizan, S. D. P. (2017). Ecovila como alternativa de organização socioambiental sustentável: uma avaliação de Piracanga, Bahia. *Sociedade & Natureza*, *29*(3), 455–467. https://doi.org/10.14393/sn-v29n3-2017-7
Fois, F. (2019). Enacting Experimental Alternative Spaces. *ANTIPODE*, *51*(1), 107–128. https://doi.org/10.1111/anti.12414
Forbis, M. M. (2016). After autonomy: the zapatistas, insurgent indigeneity, and decolonization. *Settler Colonial Studies*, *6*(4), 365–384. https://doi.org/10.1080/2201473X.2015.1090531
Gahman, L. (2017). Building 'a world where many worlds fit': Indigenous autonomy, mutual aid, and an (anti-capitalist) moral economy of the (rebel) peasant. In *Sustainable Food Futures: Multidisciplinary Solutions*. https://www.scopus.com/inward/record.uri?eid=2-s2.0-

85124014595&partnerID=40&md5=63acede27b9c7d5ac06de3cffe91913b

Gorissen, L., Spira, F., Meynaerts, E., Valkering, P., & Frantzeskaki, N. (2018). Moving towards systemic change? Investigating acceleration dynamics of urban sustainability transitions in the Belgian City of Genk. *Journal of Cleaner Production*, *173*, 171–185. https://doi.org/10.1016/j.jclepro.2016.12.052

Grasseni, C. (2014). Seeds of trust. Italy's Gruppi di Acquisto Solidale (Solidarity Purchase Groups). *Journal of Political Ecology*, *21*(1), 178–192. https://doi.org/10.2458/v21i1.21131

Hong, S., & Vicdan, H. (2016). Re-imagining the utopian: Transformation of a sustainable lifestyle in ecovillages. *Journal of Business Research*, *69*(1), 120–136. https://doi.org/10.1016/j.jbusres.2015.07.026

Javier, M., & Sastoque, M. (2012). Neoruralism As Configurative Practice of Alternative Social Dynamics: a Case Study. *Revista Luna Azul*, *34*, 113–130.

Jimenez, A., Delgado, D., Merino, R., & Argumedo, A. (2022). A Decolonial Approach to Innovation? Building Paths Towards Buen Vivir. *Journal of Development Studies*, *58*(9), 1633–1650. https://doi.org/10.1080/00220388.2022.2043281

Kingfisher, C. (2022). *Collaborative Happiness: Building the Good Life in Urban Cohousing Communities*. Berghahn Books.

Kumbamu, A. (2018). Building sustainable social and solidarity economies: Place-based and network-based strategies of alternative development organizations in India. *Community Development*, *49*(1), 18–33. https://doi.org/10.1080/15575330.2017.1384744

Lang, M. (2019). Plurinationality as a strategy: transforming local state institutions toward buen vivir. In Klein, E., & Morreo, C. E. (Eds.). *Postdevelopment in practice: Alternatives, economies, ontologies* (pp.176-189). https://doi.org/10.4324/9780429492136

Levidow, L., Sansolo, D., & Schiavinatto, M. (2021). Agroecological innovation constructing socionatural order for social transformation: two case studies in Brazil [Inovação agroecológica construindo ordem socionatural para transformação social: dois estudos de caso brasileiros]. *Tapuya: Latin American Science, Technology and Society*, *4*(1). https://doi.org/10.1080/25729861.2020.1843318

Lloveras, J., Warnaby, G., & Quinn, L. (2020). Mutualism as market practice: An examination of market performativity in the context of anarchism and its implications for post-capitalist politics. *Marketing Theory*, *20*(3), 229–249. https://doi.org/10.1177/1470593119885172

Lockyer, J. (2017). Community, commons, and degrowth at Dancing Rabbit Ecovillage. *Journal of Political Ecology*, *24*(1), 519–542. https://doi.org/10.2458/v24i1.20890

Luna, D. I. (2013). El arcoiris terrestre como universal posible desde el zapatismo. *Argumentos (México, D.F.)*, *26*(73), 109–130. http://www.scielo.org.mx/scielo.php?script=sci_arttext&pid=S0187-57952013000300007&lang=pt

Macías-Macías, A., & Sevilla-García, Y. L. (2020). Practi-torio comunidad y buen vivir: en la búsqueda de alternativas al desarrollo en el sur de Jalisco, México. *Estudios Sociales. Revista de Alimentación Contemporánea y Desarrollo Regional*, *30*(56). https://doi.org/10.24836/es.v30i56.1018

Miles, M. (2008). *Urban Utopias: The Built and Social Architectures of Alternative Settlements*. https://doi.org/10.1111/j.1468-

2427.2010.00946_6.x
Moravcikova, D., & Furjeszova, T. (2018). Ecovillage as an alternative way of rural life: Evidence from Hungary and Slovakia. *European Countryside*, *10*(4), 693–710. https://doi.org/10.2478/euco-2018-0038
Moreira, S., & Fuster Morell, M. (2020). Food Networks As Urban Commons: Case Study of a Portuguese "Prosumers" Group. *Ecological Economics*, *177*. https://doi.org/10.1016/j.ecolecon.2020.106777
Morris, O. (2022). How ecovillages work: more-than-human understandings of rentabilidad in Mexican ecovillages. *Sustainability Science*, *17*(4), 1235–1246. https://doi.org/10.1007/s11625-022-01162-7
Muñoz-Villarreal, E. M. (2018). Ecoaldeas en Colombia transitando hacia el buen vivir. *Entramado*, *14*(2), 114–131. https://doi.org/10.18041/1900-3803/entramado.2.4754
Nelson, A. (2018). Nonmonetary eco-collaborative living for degrowth. In *Housing for Degrowth: Principles, Models, Challenges and Opportunities*. https://doi.org/10.4324/9781315151205-22
Nirmal, P., & Rocheleau, D. (2019). Decolonizing degrowth in the post-development convergence: Questions, experiences, and proposals from two Indigenous territories. *Environment and Planning E: Nature and Space*, *2*(3), 465–492. https://doi.org/10.1177/2514848618819478
North, P. (2005). Scaling alternative economic practices? Some lessons from alternative currencies. *Transactions of the Institute of British Geographers*, *30*(2), 221–233. https://doi.org/10.1111/j.1475-5661.2005.00162.x
Pereira, L. M., Karpouzoglou, T., Frantzeskaki, N., & Olsson, P. (2018). Designing transformative spaces for sustainability in social-ecological systems. *Ecology and Society*, *23*(4). https://doi.org/10.5751/ES-10607-230432
Pereira, L. M., Karpouzoglou, T., Frantzeskaki, N., & Olsson, P. (2018). Designing transformative spaces for sustainability in social-ecological systems. *Ecology and Society*, *23*(4). https://doi.org/10.5751/ES-10607-230432
Pisters, S. R., Vihinen, H., Figueiredo, E., & Wals, A. E. J. (2023). 'We Learned the Language of the Tree' Ecovillages as Spaces of Place-Based Transformative Learning. *Journal of Transformative Education*, *21*(1), 59–83. https://doi.org/10.1177/15413446211068550
Price, O. M., Ville, S., Heffernan, E., Gibbons, B., & Johnsson, M. (2020). Finding convergence: Economic perspectives and the economic practices of an Australian ecovillage. *Environmental Innovation and Societal Transitions*, *34*, 209–220. https://doi.org/10.1016/j.eist.2019.12.007
Richardson-Ngwenya, P. (2021). Everyday political geographies of community-building: Exploring the practices of three Zimbabwean permaculture communities. *Environmental Policy and Governance*, *31*(3), 211–222. https://doi.org/10.1002/eet.1930
Rocha, R. S. S. (2022). Degrowth in Practice: Developing an Ecological Habitus within Permaculture Entrepreneurship. *Sustainability (Switzerland)*, *14*(14). https://doi.org/10.3390/su14148938
Rubin, Z. (2019). Ecovillagers' assessment of sustainability: Differing perceptions of technology as a differing account of modernism. *Sustainability (Switzerland)*, *11*(21). https://doi.org/10.3390/su11216167

Schöneberg, J. (2019). Manoeuvring political realms: alternatives to development in Haiti. In Klein, E., & Morreo, C. E. (Eds.). *Postdevelopment in practice: Alternatives, economies, ontologies* (pp.263-275). https://doi.org/10.4324/9780429492136

Schöneberg, J., Haudenschild, D., Darvishi, H., Momeni, S., & Ziai, A. (2022). The many faces of Post-Development: alternatives to development in Tanzania, Iran and Haiti. *Sustainability Science*, *17*(4), 1223–1234. https://doi.org/10.1007/s11625-022-01164-5

Tummers, L., & MacGregor, S. (2019). Beyond wishful thinking: a FPE perspective on commoning, care, and the promise of co-housing. *International Journal of the Commons*, *13*(1, SI), 62–83. https://doi.org/10.18352/ijc.918

Uharte, L. M. (2022). The communal economy in Venezuela from the perspective of the solidarity economy: an ethnographic approach to the communes [La economía comunal en Venezuela desde la óptica de la economía solidaria: una aproximación etnográfica a las comunas]. *AIBR Revista de Antropologia Iberoamericana*, *17*(3), 491–515. https://doi.org/10.11156/aibr.170304

Ulug, C., Horlings, L., & Trell, E.-M. (2021). Collective identity supporting sustainability transformations in ecovillage communities. *Sustainability (Switzerland)*, *13*(15). https://doi.org/10.3390/su13158148

Valencia, Ó. E., & Courtheyn, C. (2023). Peace through coca? Decolonial peacebuilding ecologies and rural development in the Territory of Conviviality and Peace of Lerma, Colombia. *Third World Quarterly*. https://doi.org/10.1080/01436597.2023.2175656

Wilson, A. D. (2013). Beyond alternative: Exploring the potential for autonomous food spaces. *Antipode*, *45*(3), 719–737. https://doi.org/10.1111/j.1467-8330.2012.01020.x
